# Near-infrared fluorescence activation probes based on disassembly-induced emission cyanine dye†
†Electronic supplementary information (ESI) available. See DOI: 10.1039/c5sc01330e



**DOI:** 10.1039/c5sc01330e

**Published:** 2015-05-25

**Authors:** Tai-Cheng Hou, Ying-Yi Wu, Po-Yi Chiang, Kui-Thong Tan

**Affiliations:** a Department of Chemistry , National Tsing Hua University , 101 Sec. 2, Kuang Fu Rd , Hsinchu 30013 , Taiwan , Republic of china . Email: kttan@mx.nthu.edu.tw ; Tel: +886-3-5715131; b Frontier Research Center on Fundamental and Applied Sciences of Matters , National Tsing Hua University , 101 Sec. 2, Kuang Fu Rd , Hsinchu 30013 , Taiwan , Republic of china

## Abstract

Currently most of the fluorogenic probes are designed for the detection of enzymes which work by converting the non-fluorescence substrate into the fluorescence product *via* an enzymatic reaction. On the other hand, the design of fluorogenic probes for non-enzymatic proteins remains a great challenge. Herein, we report a general strategy to create near-IR fluorogenic probes, where a small molecule ligand is conjugated to a novel γ-phenyl-substituted Cy5 fluorophore, for the selective detection of proteins through a non-enzymatic process. Detail mechanistic studies reveal that the probes self-assemble to form fluorescence-quenched J-type aggregate. In the presence of target analyte, bright fluorescence in the near-IR region is emitted through the recognition-induced disassembly of the probe aggregate. This Cy5 fluorophore is a unique self-assembly/disassembly dye as it gives remarkable fluorescence enhancement. Based on the same design, three different fluorogenic probes were constructed and one of them was applied for the no-wash imaging of tumor cells for the detection of hypoxia-induced cancer-specific biomarker, transmembrane-type carbonic anhydrase IX.

## Abstract 2

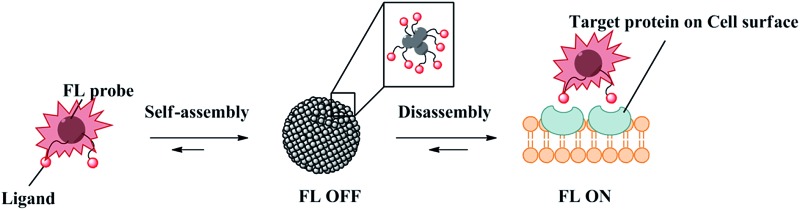
In the presence of target analyte, bright fluorescence in the near-IR region is emitted through the recognition-induced disassembly of the probe aggregate.

## Introduction

Analyte responsive fluorescence activation chemical probes (fluorogenic probes) are important tools in basic biology research and medical diagnosis because they allow for sensitive, simple and specific detection of target molecules with high signal-to-background ratios in complex environments, such as cell lysates, living cells, and *in vivo*.^1,2^ Currently, most of the fluorogenic probes are reaction-based and designed for monitoring enzyme activities and reactive small molecule species.^3–6^ Typically, their activation mechanism is based on the bond cleavage of the probe by the target analytes to produce fluorescence enhancement. Although undoubtedly valuable, this strategy is not applicable to a myriad of non-enzymatic proteins such as receptors and transport proteins which do not possess the necessary enzymatic action to disrupt the probe structure. Thus, laborious and cumbersome immunoassays are normally utilized for the detection and quantification of this class of proteins. Clearly, the establishment of a general fluorogenic probe strategy which can respond selectively to non-enzymatic targets is highly attractive, as it can provide a simple and rapid method to analyze non-enzymatic proteins in living cells or *in vivo*.

To date, the design of fluorogenic probes for simple, rapid and sensitive detection of non-enzymatic proteins remains a challenging task, as only a limited number of fluorescence activation strategies have been reported, for example, hairpin peptide beacons,^7,8^ supramolecular approaches,^9–13^ conjugates of environment-sensitive fluorophores^14–16^ or aggregation-induced emission dyes.^17^ In these designs, target proteins are recognized by the probes through the covalently attached chemical ligand or specific peptide sequence. Following the molecular recognition event, fluorescence can be turned-on by various activation mechanisms, such as Förster Resonance Energy Transfer (FRET), changes to the protein hydrophobic microenvironment, dye disassembly or aggregation-induced emission. Although these fluorogenic probes use novel strategies for the analysis of non-enzymatic proteins, nevertheless, most of them have several disadvantages, such as requiring long and elaborate synthetic steps to obtain the fluorogenic probes or have only small-to-moderate fluorescent turn-on ratios. Furthermore, all of these strategies employ fluorophores that absorb and emit in the UV-visible region. As cells also show strong autofluorescence in this region, the samples would incur high background noise which leads to low signal-to-noise ratios.^18,19^

**Fig. 1:**
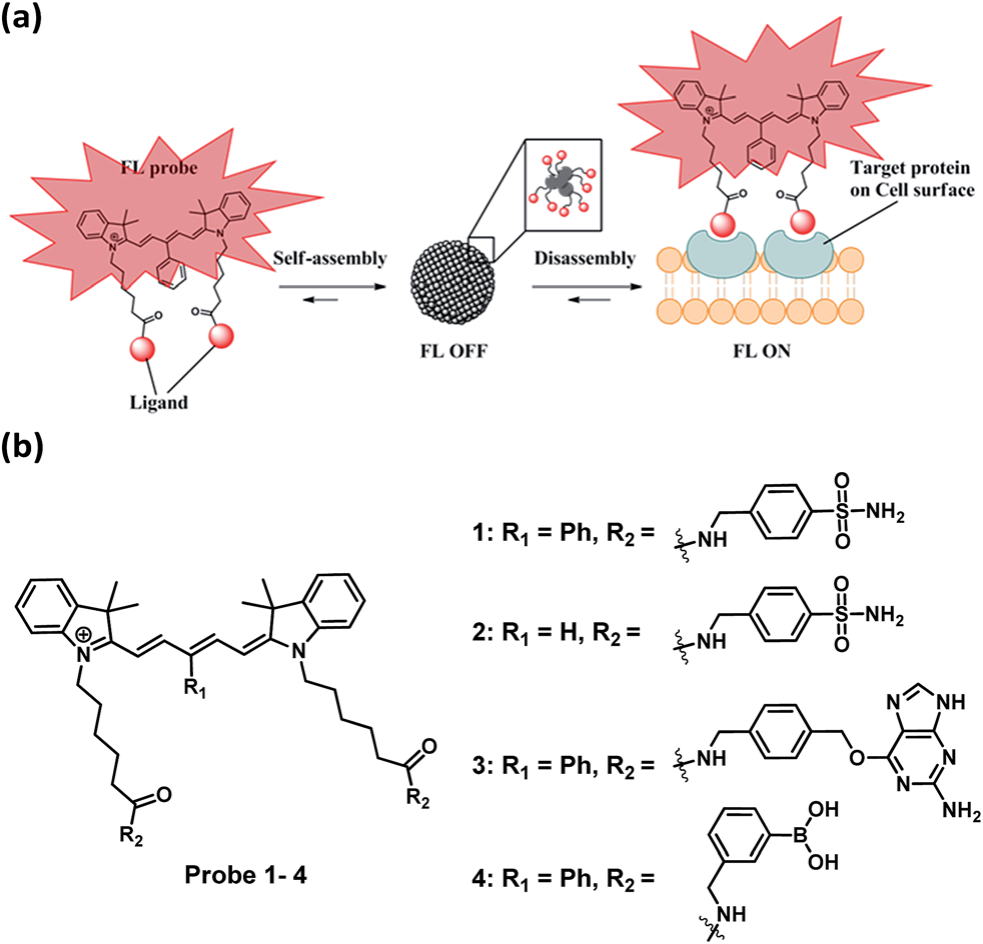
(a) Schematic illustration of dynamic near-IR fluorogenic probes based on disassembly-induced emission Cy5 dye for selective target analyte detection and the application in no-wash live cell imaging of cell surface proteins. (b) Chemical structures of fluorogenic probes. **1** and **2** for carbonic anhydrase, **3** for SNAP-tag protein, **4** for fructose.

Herein, we report a general strategy to create analyte-responsive near-infrared (near-IR) fluorogenic probes for the selective detection of proteins through a non-enzymatic process. Our probe design is based on a novel ligand-conjugated Cy5 fluorophore which functions as a protein-specific probe (Fig. 1a). In the absence of target protein, the probe forms a self-assembled aggregate which displays only weak fluorescence, whereas bright fluorescence is emitted in response to the target protein through the recognition-induced disassembly of the probe aggregate. Based on the same design, three different fluorogenic probes were constructed and one of them was applied for the no-wash imaging of tumor cells for the detection of cancer-specific biomarker, transmembrane-type carbonic anhydrase IX. In this paper, the Cy5 fluorophore employed for the creation of the fluorogenic probe consists of a γ-substituted phenyl group at the pentamethinium bridge.^20,21^ We found that this Cy5 fluorophore is a unique and optimal self-assembly/disassembly dye as it gives remarkable fluorescence enhancement in near-IR region upon recognition of the ligand to the target protein. In comparison, we observed that the classical Cy5 dye without this γ-substituted phenyl group gave no such fluorescence increase.

## Results and discussion

### Cy5-based fluorogenic probe toward carbonic anhydrase detection

**Fig. 2:**
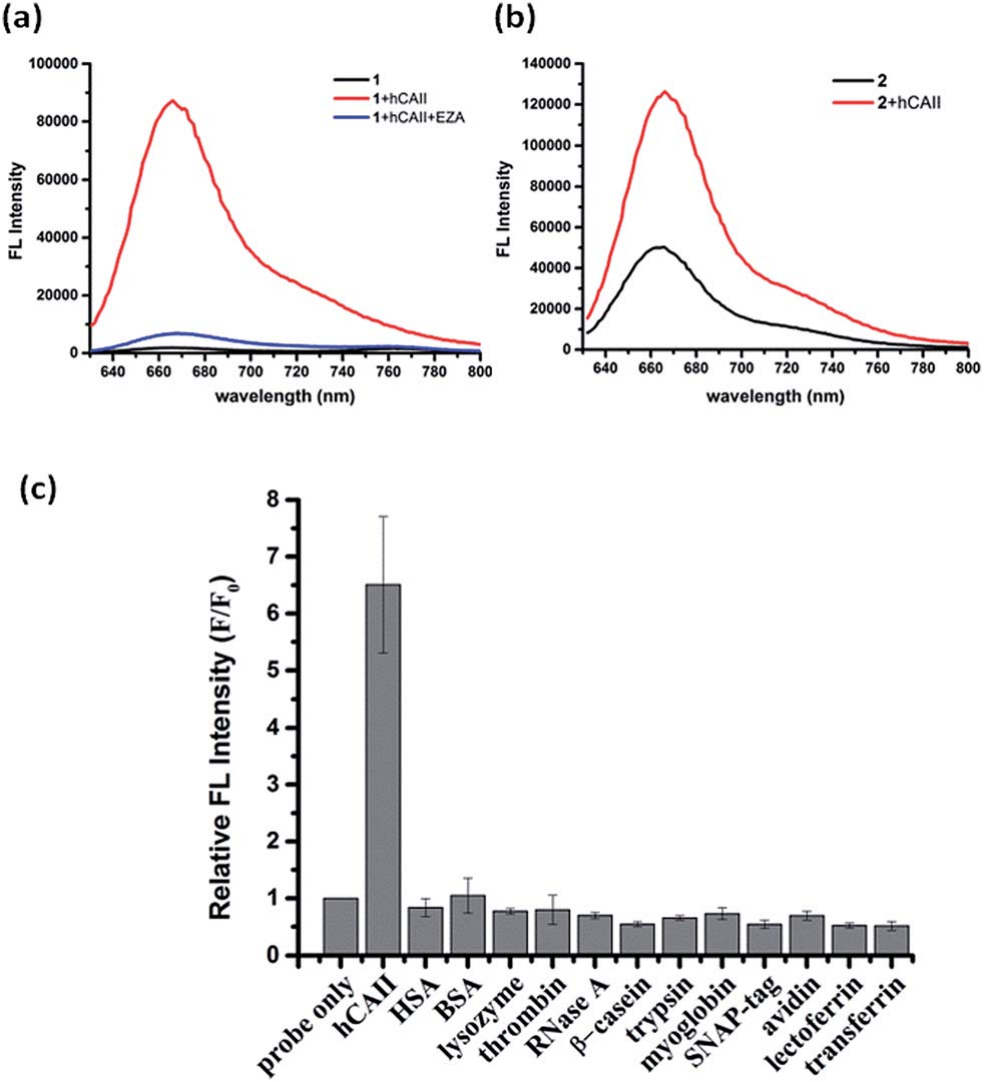
Detection of hCAII with sulfonamide probe **1** and **2**. (a) Fluorescence spectra of 5 μM **1** in the absence and presence of 10 μM hCAII in PBS buffer (1% DMSO), and after addition of 100 μM ethoxzolamide (EZA), a strong hCAII competitive inhibitor. *λ*_ex_ = 600 nm. The fluorescence turn-on ratio was calculated from the relative fluorescence intensity of **1** at *λ*_em_ = 668 nm. (b) Fluorescence response of 5 μM **2** in the absence and presence of 10 μM hCAII. (c) Selectivity test of 5 μM probe **1** with hCAII and twelve non-targeted proteins (1 μM). Error bars were calculated from three independent measurements.

To test the probe design, human carbonic anhydrase II (hCAII), a monomeric soluble protein that consists of an arylsulfonamide binding site was chosen as a target protein. hCAII and many of its isoforms are important proteins in the regulation of numerous physiological process, including pH and CO_2_ homeostasis, bone resorption, calcification, and tumorigenicity.^22^ For the detection of hCAII, probe **1** was prepared by reacting benzylamine sulfonamide with the γ-substituted Cy5 dye under the standard peptide coupling condition (Fig. 1b and Scheme S1†). Probe **1** gives extremely weak fluorescence (*ε* = 47 109 M^–1^ cm^–1^ (*λ*_max_ = 648 nm), *φ* = 0.019, Fig. 2a) in aqueous PBS buffer. However, fluorescence was enhanced dramatically in the presence of hCAII protein with a high turn-on ratio of around 48-fold (*ε* = 85 575 M^–1^ cm^–1^ (*λ*_max_ = 648 nm), *φ* = 0.28). The fluorescence of **1** can be quenched again by the addition of ethoxzolamide, a strong competitive inhibitor for hCAII. This demonstrates the dynamic fluorescence switching mechanism where fluorescence activation is controlled by the recognition of the benzenesulfonamide ligand of probe **1** by hCAII. Job's plot analysis revealed that the emission intensity of **1** peaked at 1 : 2 mole fraction of probe **1** to hCAII (Fig. S1†). The results were consistent with the chemical structure of **1** which consists of two benzenesulfonamide moieties for binding with two hCAII proteins. From the titration experiment, the limit of detection (LOD) for probe **1** to detect hCAII was determined to be as low as 17 nM which shows the high sensitivity of this fluorogenic probe (Fig. S2†).

To ascertain whether other Cy5 dyes could be used in this design, we attached the same arylsulfonamide to the classical Cy5 dye which has no γ-substituted phenyl group at the pentamethinium bridge. Interestingly, compound **2** displayed only 2-fold fluorescence enhancement in the presence of hCAII (Fig. 2b). These results indicate that the choice of Cy5 dye was crucial for the probes to form fluorescent quenching aggregates and the subsequent disassembly to produce high fluorescence in the presence of target protein. Besides the high fluorescence activation ratio and superior sensitivity, probe **1** also shows excellent selectivity toward hCAII when tested against a collection of twelve different proteins (Fig. 2c). No fluorescence increase was observed when probe **1** was mixed with non-sulfonamide binding proteins. In contrast, a strong fluorescence enhancement was obtained upon the addition hCAII. The application of this fluorogenic probe design for the detection of target protein in a complex medium was also demonstrated in urine sample, Luria–Bertani medium (LB) and Dulbecco's Modified Eagle Medium (DMEM). Probe **1** displayed weak fluorescence in these mediums, but exhibited strong fluorescence enhancement when hCAII was added (Fig. S3†). Furthermore, we also tested the performance of our probe in a medium containing high protein concentration (5% serum in PBS buffer, total protein concentration was about 1.9 g L^–1^). Fluorescence enhancement can still be obtained upon addition of 0.5 μM hCAII in spite of the higher background fluorescence (Fig. S3†). Thus, this design presents a very valuable approach for target protein detection in various complex mediums.

### Characterization of self-assembly Cy5-based fluorogenic probe

**Fig. 3:**
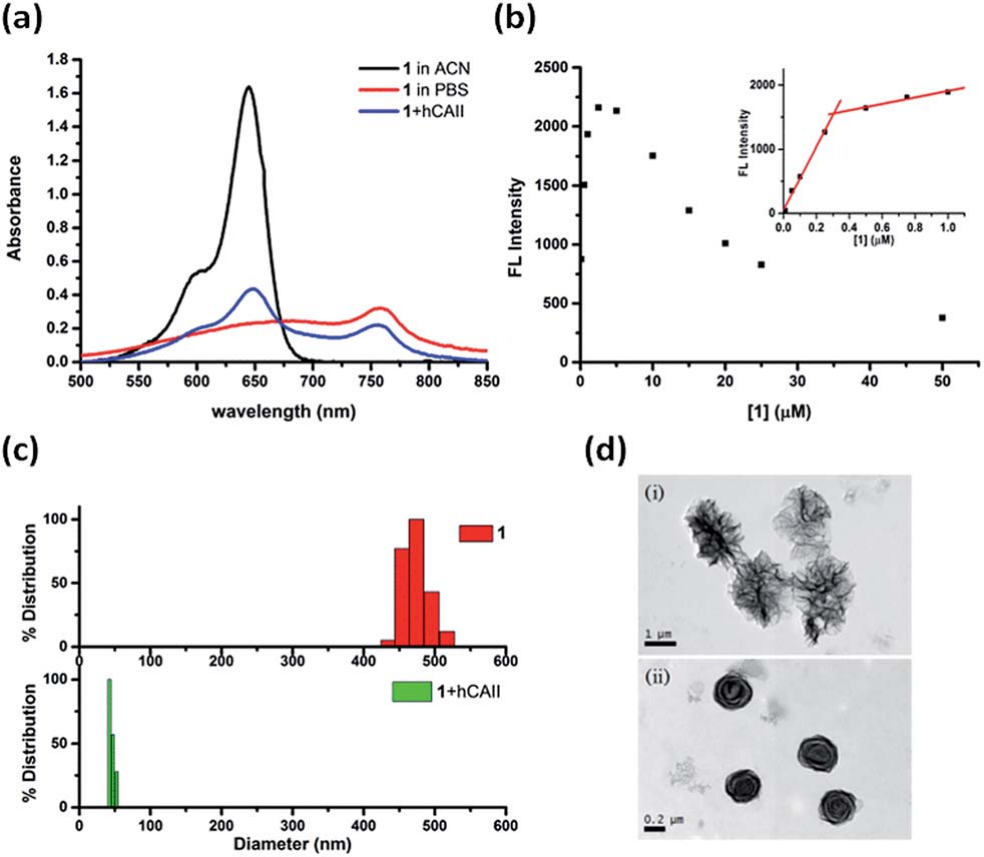
Self-assembly and disassembly analyses of hCAII probe **1**. (a) UV-vis absorption spectra of 5 μM **1** in ACN, PBS buffer, and in the presence of 10 μM hCAII. (b) Plots of the fluorescence intensity (*λ*_em_ = 668 nm) in PBS buffer with increasing **1** concentrations. The inset shows that the fluorescence intensity increases linearly up to a probe concentration of 0.25 μM. (c) DLS analyses of the particle size distribution of 5 μM **1** in the absence and presence of 10 μM hCAII. (d) TEM images of (i) 5 μM probe **1** only and (ii) the mixture of 5 μM probe **1** and 10 μM hCAII.

The weak fluorescence of **1** in PBS buffer but strong fluorescence enhancement in the presence of hCAII prompted us to study the self-assembly/disassembly properties of **1** in greater detail. First, the absorption spectra of **1** under various conditions were measured. Probe **1** dissolved in acetonitrile (ACN) showed an absorption maximum at 640 nm which is typical for many Cy5 dyes existing in monomeric state (Fig. 3a). In sharp contrast, we observed a broad absorption band and a red shift of the absorption maximum to 756 nm for **1** in PBS buffer. In the presence of hCAII, the absorption maximum of **1** was shifted back to 644 nm. The characteristic feature of the absorption spectra suggests that probe **1** in PBS buffer forms J-type aggregate which typically exhibits red-shifted absorption maximum with respect to that of the monomer.^23–25^ Addition of hCAII disrupts the aggregation and the red-shifted absorption maximum of **1** at 756 nm gradually decreases while the absorption band at 644 nm increased with rising hCAII concentration (Fig. S4†). In agreement with the formation of fluorescence-quenched J-aggregate, a substantial decrease in the fluorescence was observed with increasing probe **1** concentration in PBS buffer (Fig. 3b). The fluorescence quenching due to aggregation occurs at the concentrations as low as 0.25 μM (Fig. 3b, inset). In comparison, compound **2** exhibits red-shifted absorption J-band and fluorescence quenching at a relatively high concentration of 10 μM (Fig. S5†). However, even at this concentration, fluorescence of compound **2** shows only about 4-fold enhancement in the presence of hCAII (Fig. S6†).

The self-assembly/disassembly property of probe **1** was further investigated with dynamic light scattering (DLS) and transmission electron microscope (TEM). DLS measurements of **1** showed aggregates with a mean diameter of around 470 nm in PBS buffer solution, whereas particle sizes of **1** was decreased to around 40 nm after addition of hCAII to the solution (Fig. 3c). The aggregates of probe **1** can be disassembled completely when a higher concentration of hCAII (20 μM) was added to the probe **1** solution (Fig. S7†). We also found that the particle size of free probe **1** is concentration-dependent. At 10 μM concentration, the particle size of probe **1** was increased to 1353 nm (Fig. S8†), while at 1 μM concentration, the particle size was reduced to around 100 nm. TEM revealed the formation of spherical particles of **1** with diameters ranging from 1 μm to 1.5 μm (Fig. 3d). In the presence of hCAII, the large aggregates observed by TEM were reduced to smaller particles with diameters ranging from 0.1 μm to 0.3 μm. These data correlate very well with the absorption and emission spectra and strongly supports the proposed mechanism where probe **1** forms self-assembled fluorescence-quenched J-type aggregates which undergoes disassembly upon binding of the benzenesulfonamide ligand of **1** to hCAII, resulting in the fluorescence activation of the Cy5 dye.

### Self-assembly Cy5-based fluorogenic probes for SNAP-tag protein labeling and small molecule sensing

To demonstrate the modular nature of our fluorogenic probe design, we replaced the benzenesulfonamide on the Cy5 dye with O6-benzylguanine (BG) to generate probe **3** for the fluorescence activation labeling of SNAP-tag protein (Fig. S9†). SNAP-tag is one of the most prominent labeling tools in cell biology and is well-known for rapid reaction rate with BG derivatives and non-toxicity in cells.^26^ Covalent labeling of SNAP-tag with an affinity ligand or optical probe has been used for protein interaction studies,^27^ drug discovery,^28^ super-resolution imaging applications,^29^ and the construction of fluorescent sensors.^30,31^ Although many fluorogenic probes for SNAP-tag have been reported, they generally suffer from either slow labeling rate, low-to-moderate fluorescence turn-on ratios, or require UV-visible light for excitation.^32–35^

**Fig. 4:**
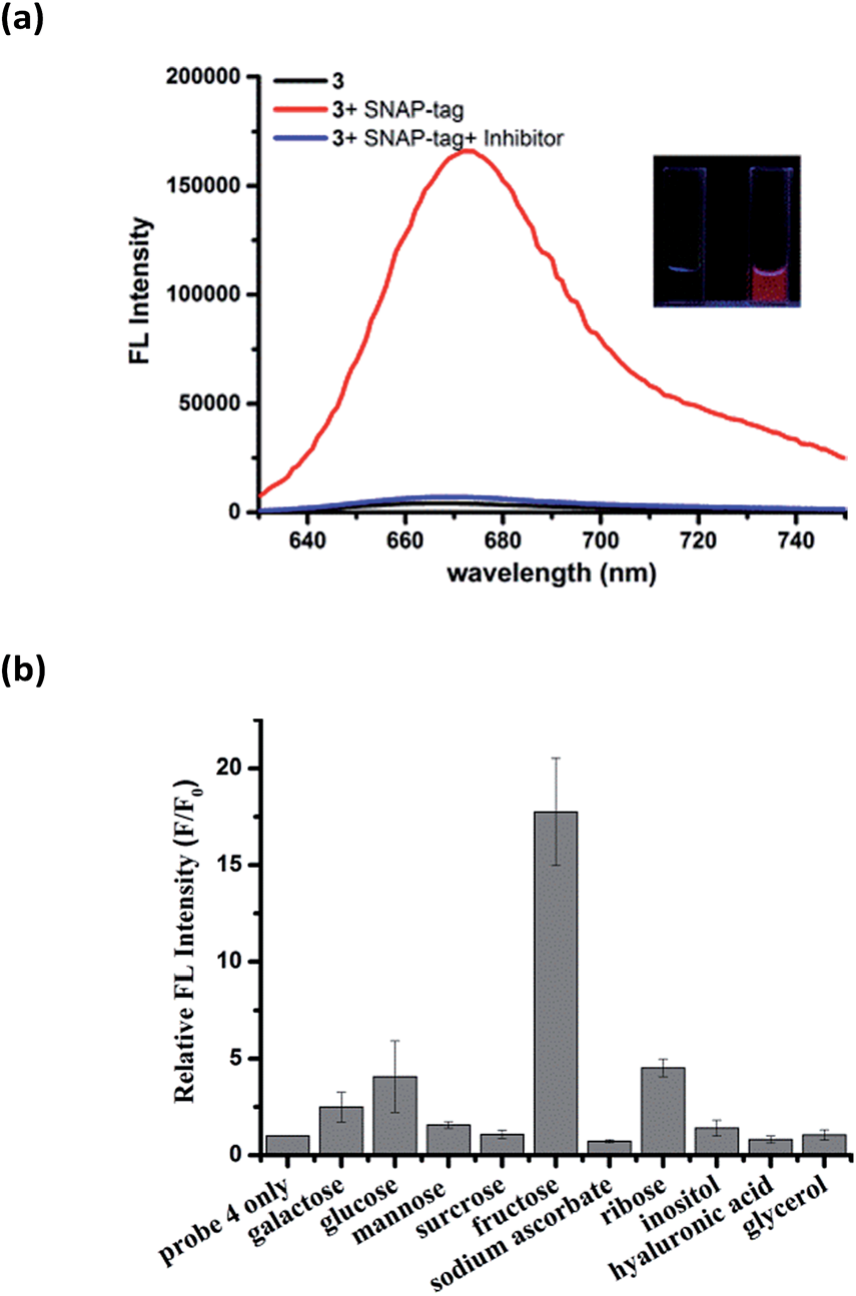
Fluorescence response of probe **3** and **4** with SNAP-tag and fructose. (a) Fluorescence spectra of 5 μM **3** in the absence and presence of 10 μM SNAP-tag protein and 100 μM O6-benzylguanine (BG). The inset shows the images of the probe **3** solution in a cuvette before (left) and after (right) addition of SNAP-tag under excitation with a UV lamp (365 nm). (b) Selectivity test of 10 μM probe **4** with 5 mM of different *cis*-diols in pH 10 PBS buffer. The concentration of hyaluronic acid was 0.5 mg mL^–1^.

Similar to the hCAII probe **1**, probe **3** displayed very weak fluorescence in PBS buffer. Upon addition of SNAP-tag protein, the fluorescence was enhanced dramatically by 40-fold (Fig. 4a). Fluorescence enhancement was so significant that it can be observed easily under handheld UV lamp (Fig. 4a, inset). The fluorescence enhancement was reduced dramatically when the inhibitor O6-benzylguanine (O6-BG, 100 μM) was pre-incubated with SNAP-tag protein. The specificity of probe **3** was also investigated by incubating **3** with thirteen other non-target proteins. In all cases, dramatic fluorescence increase was observed only when SNAP-tag was present which demonstrates the specific interaction between probe **3** and SNAP-tag (Fig. S10†). From Job's plot analysis, absorption spectra and DLS measurements of **3** in the absence or presence of SNAP-tag (Fig. S11†), we concluded that the fluorescence activation mechanism of **3** also involves the formation of fluorescence-quenched J-type aggregates, and that the disassembly of the aggregates triggered by SNAP-tag interaction leads to the fluorescence activation of probe **3**. We also found that the reaction kinetics of probe **3** with SNAP-tag was not deterred by the formation of self-assembly aggregates. A kinetic analysis of the SNAP-tag labeling reaction with **3** revealed that the time required to achieve full labeling was around four minutes with *k*_2_ of about 5930 M^–1^ s^–1^ (Fig. S12†), which is comparable to other fast SNAP-tag labeling probes.^32^

As both hCAII and SNAP-tag are macromolecules, we also tested the applicability of our design to detect small molecules by the same mechanism. To this end, we incorporated a boronic acid moiety to the γ-substituted phenyl Cy5 dye to generate probe **4** for the analysis of carbohydrates. Boronic acids can interact reversibly with *cis*-diol molecules to form boronate ester compounds. As expected, probe **4** displayed very weak fluorescence in aqueous buffer, while strong fluorescence enhancement of around 18-fold was achieved upon the addition of 5 mM fructose (Fig. 4b). Among the ten *cis*-diols studied, **4** exhibited selective binding to fructose with the highest corresponding fluorescence turn-on ratio which is consistent with the high affinity of fructose with boronic acids as reported in the literatures.^36^ The LOD for **4** to detect fructose was determined to be as low as 10 μM which puts probe **4** as one of the most sensitive fructose fluorogenic probe reported (Fig. S13†).^37^ The fluorescence activation mechanism of **4** was further affirmed by the absorption spectra and DLS which also shows that **4** forms J-aggregates in aqueous solution and the addition of fructose triggers the disassembly of J-aggregates (Fig. S14†). In the absence of target analytes, the absorption spectra of probe **3** and **4** showed maximum absorption at around 705 nm and 690 nm, respectively (Fig. S11b and S14a†). The difference between the maximum absorption for the three probes is due to the different ligands attached to the Cy5 fluorophore.

### No-wash live-cell imaging of hCAII and SNAP-tagged proteins on cell surface with disassembly-induced emission Cy5 probes

**Fig. 5:**
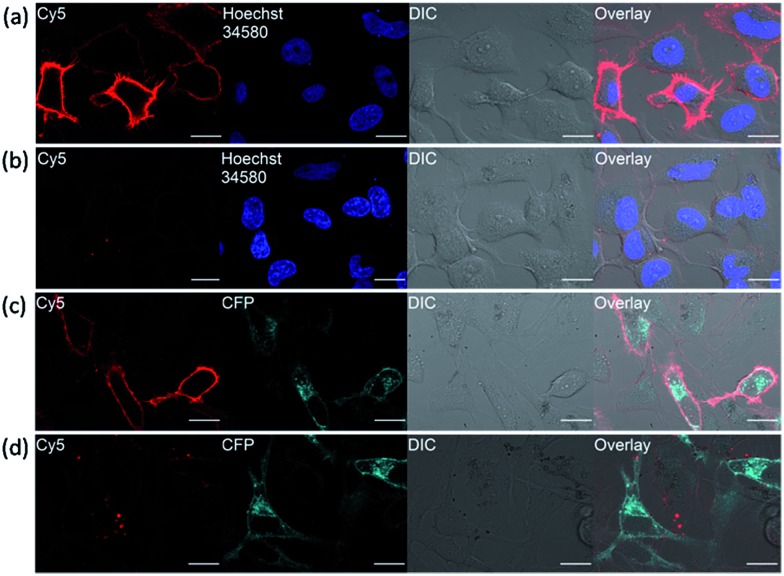
No-wash live-cell imaging of HeLa cells expressing hCAII proteins on cell surface. (a) HeLa cells expressing hCAII–PDGFR proteins on cell surface were treated with 0.5 μM probe **1** and (b) upon addition of 100 μM ethoxzolamide. (c) HeLa cells expressing CFP–hCAII–PDGFR protein were treated with 0.5 μM **1** and (d) upon addition of 100 μM ethoxzolamide. Images were taken with identical microscope setup. Scale bar: 20 μm.

In fluorescence live-cell imaging, an extensive washout procedure to remove excess unbound probe is a prerequisite for protein visualization and quantification. However, this washing step may also remove the probe from the protein, rendering the continuous monitoring of the dynamics of proteins virtually impossible. To further establish the utility of our fluorogenic probes for live cell imaging, we applied probe **1** to detect carbonic anhydrase expressed on cell surface. As a model system, the hCAII gene encoding construct was fused to the N-terminal of a transmembrane anchoring domain of platelet-derived growth factor receptor, hCAII–PDGFR, to express the protein on the cell surface. Live-cell images of HeLa cells transiently transfected with the hCAII–PDGFR plasmid were taken after probe **1** was added to the cells without any washout process. Strong fluorescence along the plasma membrane of cells expressing hCAII–PDGFR was observed using the confocal laser scanning microscope (CLSM), while non-transfected cells showed no fluorescence on the cell surface (Fig. 5a). When **1** and hCAII inhibitor ethoxzolamide (100 μM) were added to the transfected HeLa cells, a dramatic reduction in the Cy5 fluorescence was observed (Fig. 5b). To further validate that the strong fluorescence observed on the cell surface is due to the specific binding of **1** with hCAII on the cell surface, we fused a cyan fluorescent protein (CFP) gene to hCAII to construct CFP–hCAII–PDFGR plasmid. The fluorescence from the Cy5 channel overlaid very well with the CFP–hCAII–PDFGR protein expressed on the plasma membrane (Fig. 5c). In contrast, addition of ethoxzolamide reduced the Cy5 fluorescence while the emission from the CFP channel was not affected (Fig. 5d). These results indicate that **1** can specifically detect hCAII expressed on the extracellular membrane. Besides the detection of hCAII with **1** on the cell surface, probe **3** was also used for the visualization of SNAP–PDGFR proteins expressed on the HeLa cell surface with no-wash protocol. Again, clear fluorescence was observed along the plasma membrane (Fig. S15†). Thus, we believe that probe **3** can be a very useful tool in chemical biology for methods involving SNAP-tag labeling technology. As our fluorogenic probes detect only extracellular surface target proteins and require no-washing operations, this is a strong advantage over visualizing membrane proteins by auto-fluorescent proteins where strong fluorescence can also be observed in intracellular secretory pathways (Fig. 5c and d, CFP channel). Hence, our probe design presents an imaging method with high spatial and temporal resolution to study organization and function of membrane proteins.

### Tumor cells imaging by detecting hypoxia-induced transmembrane-type carbonic anhydrase IX

**Fig. 6:**
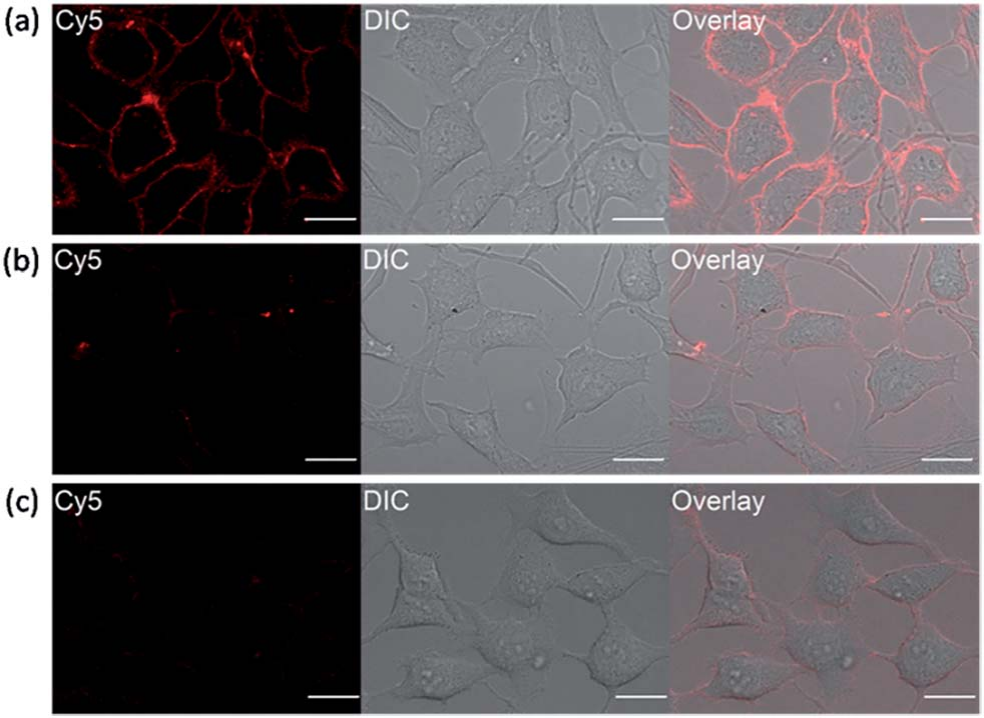
No-wash imaging of hypoxia-induced transmembrane-type carbonic anhydrase IX of HeLa cells with 0.5 μM probe **1**. (a) HeLa cells cultured under hypoxia condition, (b) upon addition of 100 μM ethoxzolamide inhibitor and (c) under normoxic conditions. All cellular images were taken on the same day with identical microscope setup. Scale bar: 20 μm.

It has been reported that transmembrane-type human carbonic anhydrase IX is substantially expressed under the condition of hypoxia in many tumor cell lines.^22^ Tumor hypoxia is associated with impaired efficacy of cancer therapies which makes hCAIX a valuable biomarker for preclinical and diagnostic imaging.^38,39^ To show that our fluorogenic probes is sensitive enough to detect endogenous proteins, probe **1** was applied for the imaging of HeLa cells, which over-express hCAIX on the extracellular surface when the cells were cultured under hypoxia condition.^40–42^ Three sets of HeLa cells were cultured for 24 hours under hypoxia (<0.1% O_2_), hypoxia-mimetic (in the presence of deferoxamine mesylate (DFO)) and normoxic conditions (20% O_2_) respectively. When probe **1** was added to the HeLa cells cultured under hypoxia condition, strong fluorescence along the cell surface was observed without washing (Fig. 6a). In contrast, weaker fluorescence was observed when the cells were cultured under normoxia conditions or when ethoxzolamide was added to the cells (Fig. 6b and c). Similar clear fluorescence images were acquired from the cells cultured under hypoxia-mimetic conditions (Fig. S16†). In addition, negligible fluorescence was observed from HEK293 cells which do not express transmembrane-type hCAIX proteins under hypoxia condition (Fig. S17†).^9^ These results clearly indicate that probe **1** is capable of sensing hypoxia environment change of cancer cells based on the over-expression level of the biomarker hCAIX. We have also conducted a western-blot analysis to validate the results from the imaging experiments (Fig. S18†). Results from the Western-blot analysis correlated very well with the fluorescence images obtained using probe **1** in which HeLa cells cultured under hypoxia-induced and hypoxia-mimic conditions gave significant Western-blot bands, while no obvious band was observed for HEK293 cells and HeLa cells cultured under normoxic conditions.

## Conclusion

In conclusion, we have developed a general design of fluorogenic probes that can be applied for the detection of target analytes in test tubes as well as the imaging of endogenous protein biomarkers under live cell no-wash conditions. The design of these near-IR fluorogenic probes is based on the novel self-assembled fluorescence-quenched Cy5 aggregates, which undergo recognition-induced disassembly in response to the target analyte to emit strong fluorescence. The modular approach greatly expands the scope of fluorogenic probes design for the detection of non-enzymatic proteins and small molecules. The probes permit specific visualization of cell surface proteins as demonstrated by the detection of hypoxia-induced endogenous carbonic anhydrase on cancer cells and SNAP-tag protein, without needing any washing operations. As compared with existing fluorescence turn-on strategies to detect non-enzymatic proteins, our near-IR probes can be prepared *via* simple synthetic steps and shows remarkable and selective fluorescence enhancement in the presence of the target protein even in complex mediums. We believe that with further progress on our fluorogenic probe design, it will be useful for a wide range of applications, such as diagnosis and live-cell imaging where high fluorescent turn-on ratios and simple detection methods are required.
