# Supplementary material for: Near-infrared fluorescence activation probes based on disassembly-induced emission cyanine dye
Source: Chem Sci. 2015 May 25;6(8):4643–9. doi: 10.1039/c5sc01330e (PMC5500852; doi:10.1039/c5sc01330e)
Supplement: Supplementary file 1 [file SC-006-C5SC01330E-s001.pdf]

Electronic Supplementary Material (ESI) for Chemical Science.
This journal is © The Royal Society of Chemistry 2015

SUPPORTING INFORMATION

Near-Infrared Fluorescence Activation Probes based on Disassembly-Induced Emission Cyanine Dye

Tai-Cheng Hou,[a] Ying-Yi Wu,[a] Po-Yi Chiang,[a] and Kui-Thong Tan [a,b,*]

a) Department of Chemistry, National Tsing Hua University, 101 Sec. 2, Kuang Fu Rd, Hsinchu 30013, Taiwan (ROC)

b) Frontier Research Center on Fundamental and Applied Sciences of Matters, National Tsing Hua University, 101 Sec. 2, Kuang Fu Rd, Hsinchu 30013, Taiwan (ROC)

Corresponding author: Kui-Thong Tan, Email: kttan@mx.nthu.edu.tw

Materials and instruments

Chemicals and reagents were purchased from Sigma-Aldrich and TCI and were used without further purification. All solvents were used after appropriate distillation or purification. Besides the hCAII and SNAP-tag protein which were expressed and purified in the laboratory, all other proteins used in the selectivity test were purchased from Sigma-Aldrich. PBS buffer (0.9 mM KCl, 2.67 mM KH_2PO_4, 138 mM NaCl, 8.1 mM Na_2HPO_4) was diluted 10-times from commercially available concentrates supplied by Amersco. Thin layer chromatography (TLC) was performed on TLC-aluminum sheets (Silica gel 60 F254, Merck). Flash column chromatography was performed with silica gel (230-400 mesh, Merck). HPLC analysis was performed with analytical column (EC 150/4.6 Nucleosil 300-5 C18, Macherey-Nagel). Products were purified by semi-preparative column (VP 150/21 Nucleosil 300-5 C18, Macherey-Nagel).

^{1}H, and ^{13}C nuclear magnetic resonance (NMR) spectra were recorded either on Varian MR-400, Varian Unityinova-500, Mercury-400 with ^{1}H chemical shifts (δ) reported in ppm relative to the solvent residual signals of d-methanol (3.30 ppm), d-DMSO (2.49 ppm). ^{13}C chemical shifts (δ) were reported in ppm relative to the solvent residual signals of d-methanol (49.0 ppm) and d-DMSO (39.5 ppm). Coupling constants were reported in Hz. Absorption spectra were recorded on Ocean Optics USB4000 spectrophotometer and using long wavelength pass filter (LAMBDA GG-400-25.4). Fluorescence spectra were recorded using Hitachi F-4500 fluorescence spectrophotometer and TECAN Infinite M200Pro. High resolution mass spectra (HRMS) were recorded on Varian 901-FTMS.

SNAP-tag and hCAII protein expression and purification

Plasmid pET51b-SNAP and pET51b-HCA both with C-terminal His-tag were transformed to *E. coli* strain BL21. The bacteria were cultured at 37 °C in LB broth medium to OD_{600nm} of 1.2. Protein expression was induced by adding 1 mM IPTG. The bacteria were grown for an additional 16 hours at 18 °C and harvested by centrifugation. The cells were lysed by sonication and insoluble protein and cell debris were removed by centrifugation. The SNAP-tag and hCAII protein were then purified by Ni-NTA. The purified protein was concentrated and transferred in PBS buffer using Amicon® Ultra centrifugal filters and snap frozen in liquid nitrogen before being stored at -78 °C. The concentration of protein was determined using BCA assay. The purity of the protein was checked by SDS-PAGE and stained by Instant Blue.

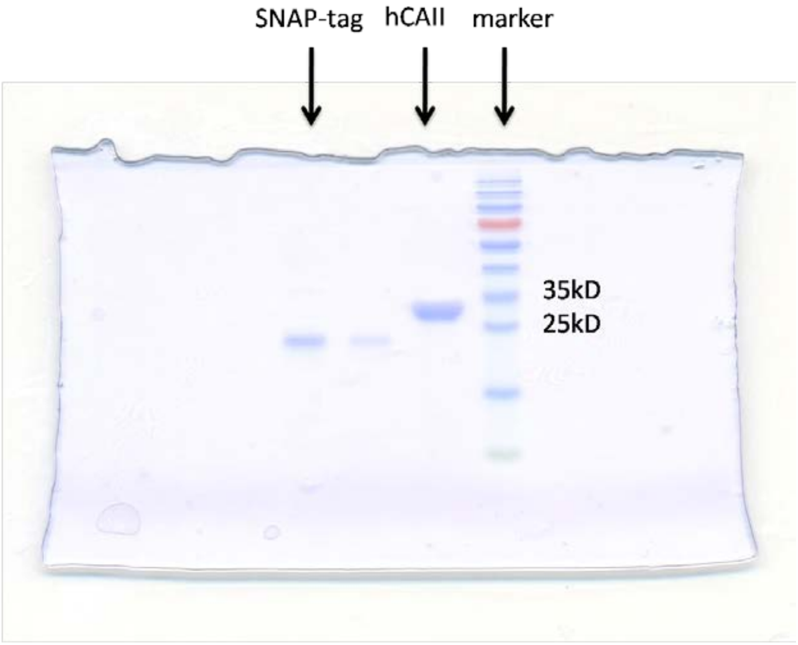

Fluorescence imaging of transfected HeLa Cells

The cells were maintained in DMEM supplemented with 10% FBS and 1% penicillin-streptomycin. 1.5×10^4 cells were seeded in 8-well chamber slides and cultured overnight at 37 °C in air with 5% CO_2. Cells were then transfected by using X-treme GENE HP DNA transfection reagent (Roche Applied Science) according to the manufacturer's protocol. Thirty hours after transfection, the cells were washed with DMEM supplemented with 10% FBS twice and cultured overnight. The cells were washed twice with Opti-MEM and stained with 0.5 μM Hoechst 34580. After thirty minutes incubation, excess Hoechst 34580 was removed. The cells were treated with 0.5 μM of probe prepared in Opti-MEM (1.0 % DMSO (v/v)). The fluorescence imaging was carried out by using Laser Scanning Confocal Microscope (LSM 700, Zeiss, Germany). For Cy5 channel, the images were taken by using 639 nm laser and LP640 emission filter. For Hoechst, we used 405 nm laser and SP490 emission filter. For CFP, we used 405 nm laser and SP555 emission filter.

Fluorescence imaging of HeLa cells under hypoxia condition

The cells were maintained in DMEM supplemented with 10% FBS and 1% penicillin-streptomycin. 2×10^4 cells were seeded in 8-well chamber slides and cultured 24 hours at 37 °C in air with 5% CO_2. The cells were cultured for 24 hours at 37°C in Opti-MEM under hypoxic conditions (<0.1% O_2) generated with an Anaero Pack (Mitsubishi Gas Chemical Company Inc.). For hypoxia-mimetic conditions, the cells were cultured in Opti-MEM with 200 μM DFO for 24 hours at 37 °C in air with 5% CO_2. The cells were imaged in Opti-MEM with the addition of 0.5 μM probe**1** (1% DMSO (v/v)) in the absence or presence of 100 μM EZA (1% DMSO (v/v)). The images were taken after staining without any washing operations.

Transmission electron microscopy (TEM)

Sample (5 μM **1** or with 10 μM hCAII) were prepared by drop casting onto 200 mesh carbon-coated copper grids and air-dried overnight. The samples were washed with water before measurement. TEM imaging was carried out with JEOL-2100 microscope with an accelerating voltage of 160 kV.

Dynamic light scattering (DLS)

DLS analyses were performed on Brookhaven 90Plus at 25℃ in PBS buffer using a plastic cuvette (3 mL volume). At least six measurements were taken. All samples were incubated for 15 minutes before measurement.

Western blot

Cell extracts were prepared by washing cells with PBS buffer and solubilizing 5×10^7 cells in 100 µL of RIPA buffer (150 mM NaCl, 1.0% NP-40, 0.5% sodium deoxycholate, 0.1% SDS, 50 mM Tris, pH 8.0). After incubation for 15 minutes on ice, the lysates were centrifuged at 12,000 rpm for 20 min at 4 °C and the supernatant was collected. 16 µL/lane of cell lysate was loaded to 10% SDS-PAGE gel. Following electrophoretic transfer of proteins onto poly(vinyl difluoride) membranes, the membranes were blocked with 5% nonfat dry milk in PBST buffer (138 mM NaCl, 2.68 mM KCl, 1.76 mM KH_2PO_4, 10.14 mM Na_2HPO_4, 0.5% tween 20, pH 7.4). The membranes were washed and then incubated with anti-hCAIX antibody (0.2 µg/mL, from Santa Cruz, CA, USA) at 4 °C overnight. After three 10-min wash with PBST buffer, the membranes were incubated with a secondary antibody (anti-mouse IgG, 0.2 µg/mL) for 1 hr. The membranes were washed before being visualized by 4CN PLUS chromogenic substrate (Perkin Elmer).

Determination of probe 1 quantum yields

The quantum yields of **1** in the absence or presence of hCAII were determined by comparing the integrated area of the corrected emission spectrum of Probe **1** with a Cy5 reference (ϕ =0.28 in PBS buffer). The quantum yield can be calculated using the following equation:

$$\Phi = \Phi_R \left(\frac{m}{m_R}\right)\left(\frac{n^2}{n^2{}_R}\right)$$

Where

m = the slope of the line obtained from the plot of the integrated fluorescence

intensity versus absorbance at 600 nm.

m_R= the slope of the line obtained from the plot of the integrated fluorescence

intensity versus absorbance of reference at 600 nm.

n = the solvent refractive index.

n_R = the solvent refractive index of reference Cy5.

ϕ = quantum yield of **1**.

ϕ_R= quantum yield of Cy5 reference.

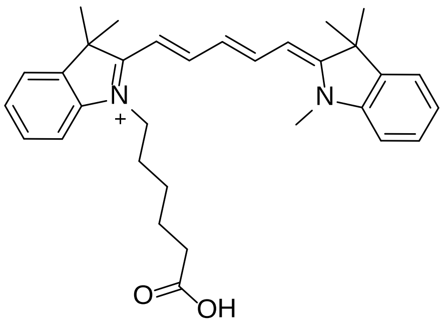

Cy5 reference (K. Umezawa, A. Matsui, Y. Nakamura, D. Citterio and K. Suzuki, *Chem. Eur. J.* 2009, **15**, 1096-1106).

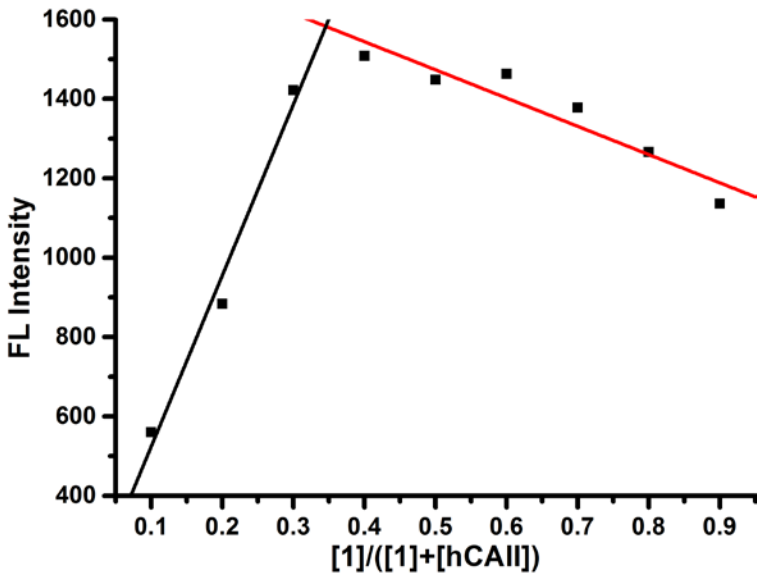

Fig. S1. Job's plot analysis of probe **1** with hCAII.

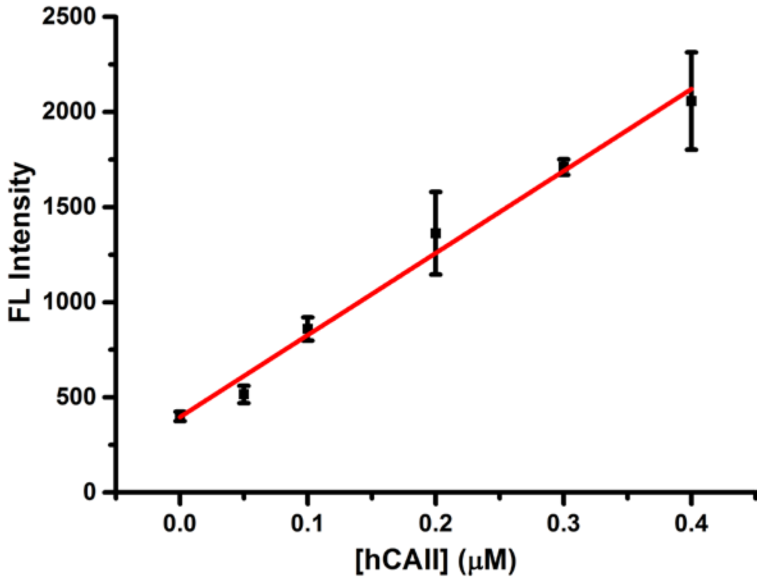

Fig. S2. Limit of detection (LOD) for probe **1** to detect hCAII concentrations. The LOD was calculated from three times the standard deviation of background. The LOD is about 17 nM. For the calculation of LOD, we used the following equation, LOD = mean fluorescent signal of blank (without target protein) + three times the standard deviation corresponding to the blank controls (N = 10).

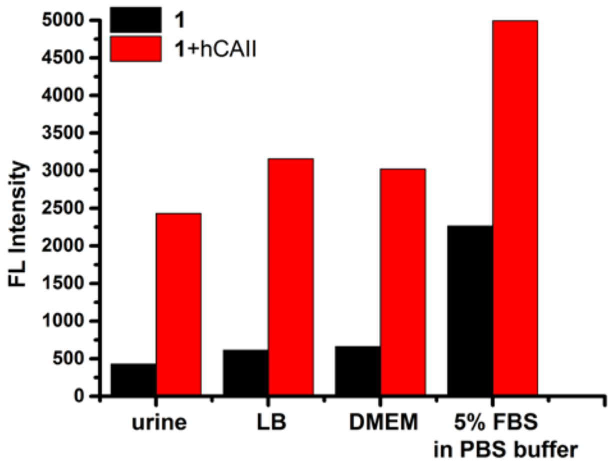

Fig. S3. Fluorescence intensity of **1** (5 μM) in the absence or presence of 0.5 μM hCAII in the indicated mediums. λ_{ex} = 600 nm, λ_{em}= 668 nm. FBS used is fetal bovine serum.

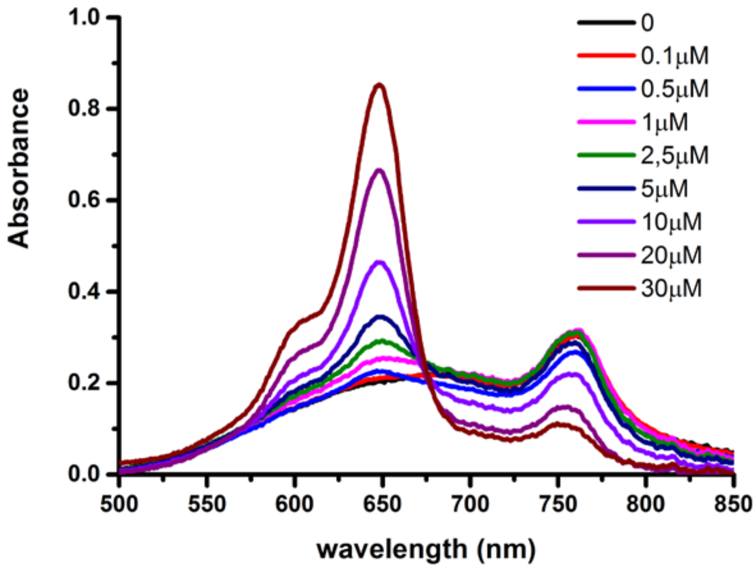

Fig. S4. Absorption spectra of 5 μM **1** with increasing hCAII concentrations.

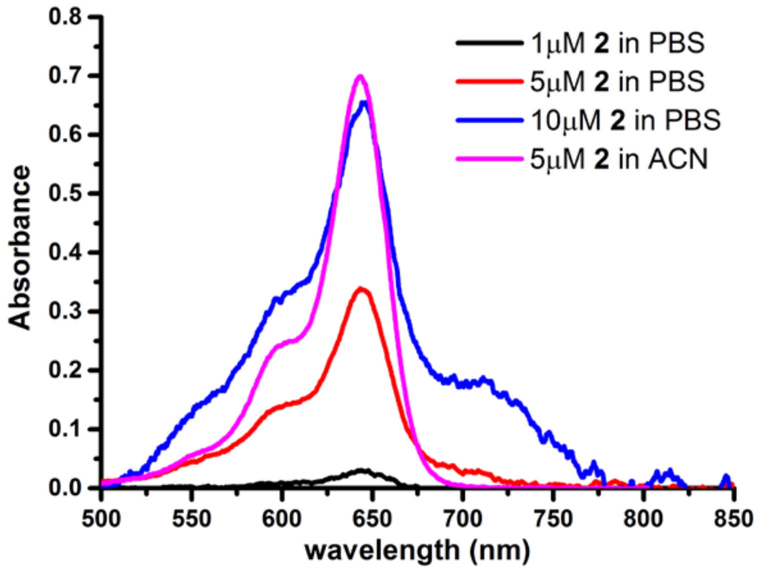

Fig. S5. Absorption spectra of compound **2** with different concentrations in PBS and CAN.

(a)

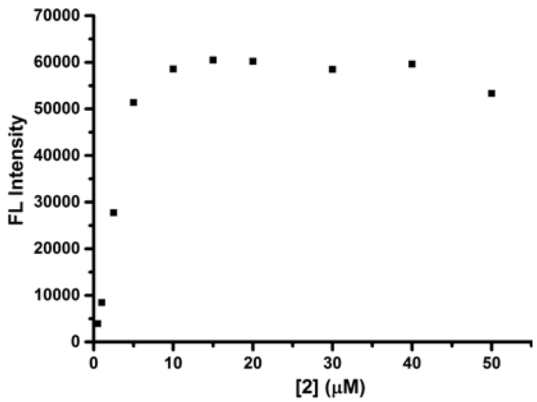

(b)

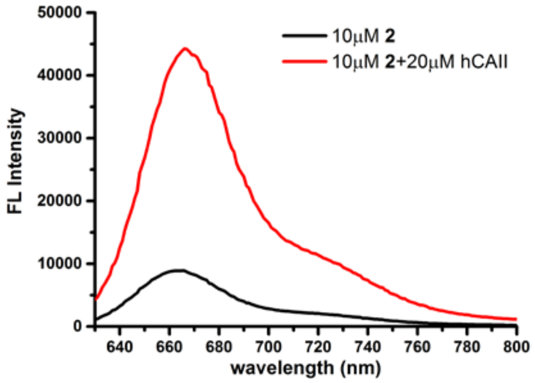

Fig. S6. (a) Plots of the fluorescence intensity (λ_{em}= 668 nm) with increasing concentrations of compound **2** in PBS buffer. (b) Fluorescence spectra of 10 μM compound **2** in the absence or presence of 20 μM hCAII. The fluorescence enhancement ratio was around 4.5-fold.

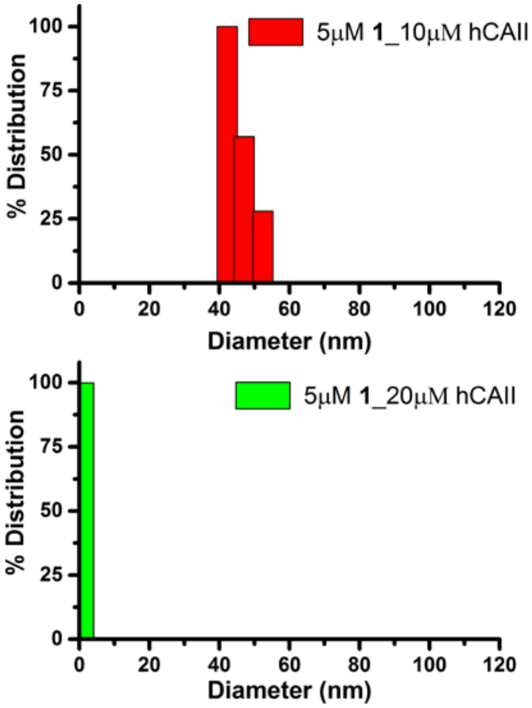

Fig. S7. DLS analyses of the particle size distribution of 5 μM **1** in the presence of 10 and 20 μM hCAII. Probe **1** was completely disassembled (<1.5 nm) in the presence of 20 μM hCAII.

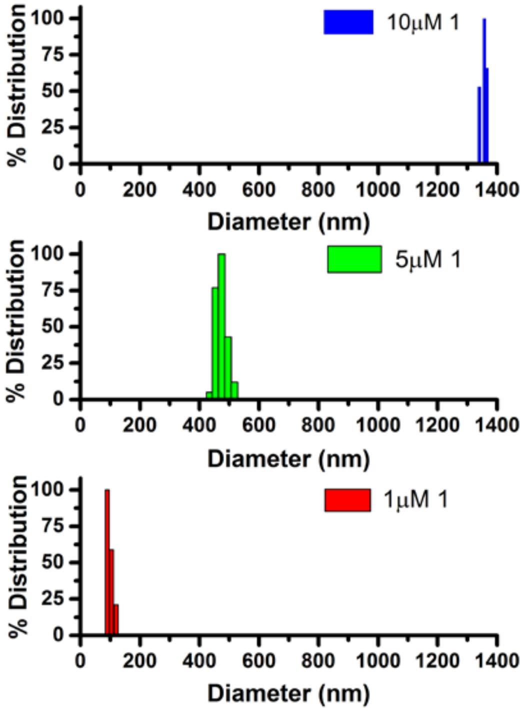

Fig. S8. DLS analyses of the particle size distribution of different probe **1** concentrations in PBS buffer.

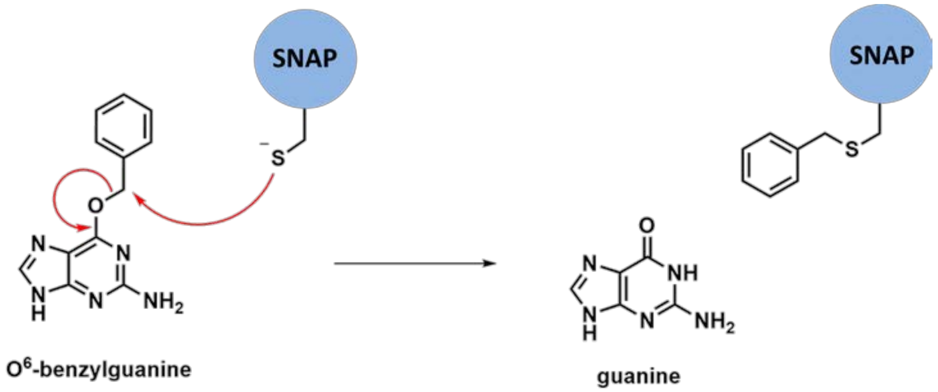

Fig. S9. Reaction mechanism of SNAP-tag protein with O6-BG compound.

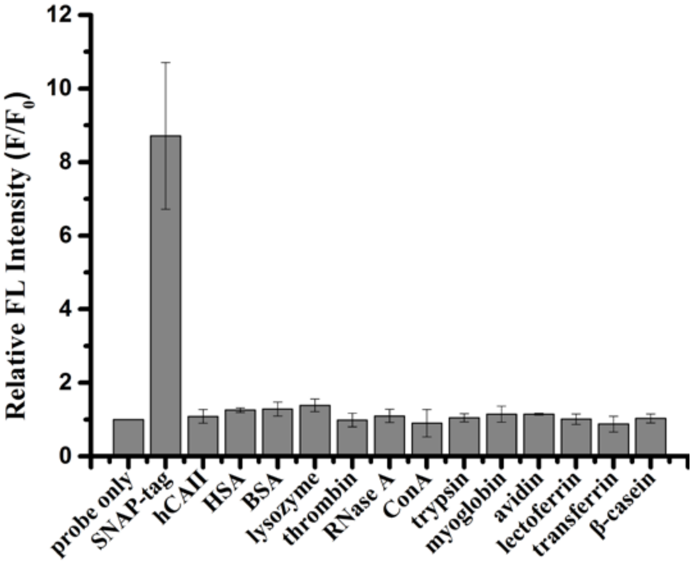

Fig. S10. Selectivity test of **3** (5 μM) with SNAP-tag and thirteen non-target proteins (1 μM).

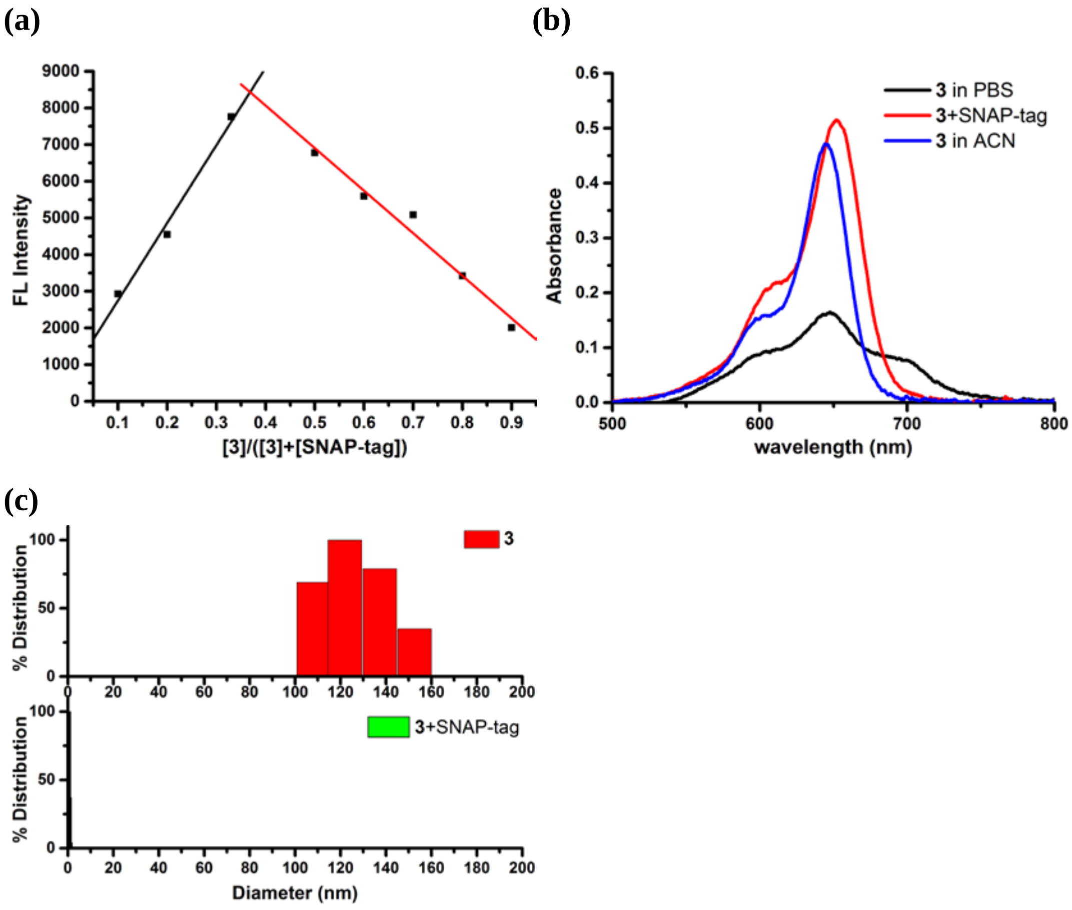

Fig. S11. (a) Job's plot analysis of probe **3** with SNAP-tag. (b) Absorption spectra of 5 μM probe **3** in ACN, PBS or with the addition of 10 μM SNAP-tag. (c) DLS analyses of the particle size distribution of 5 μM probe **3** and in the presence of 10 μM SNAP-tag. The mean diameter of **3** is about 130 nm. Negligible particle size (<1.5 nm) was observed in the presence of SNAP-tag.

(a) (b)

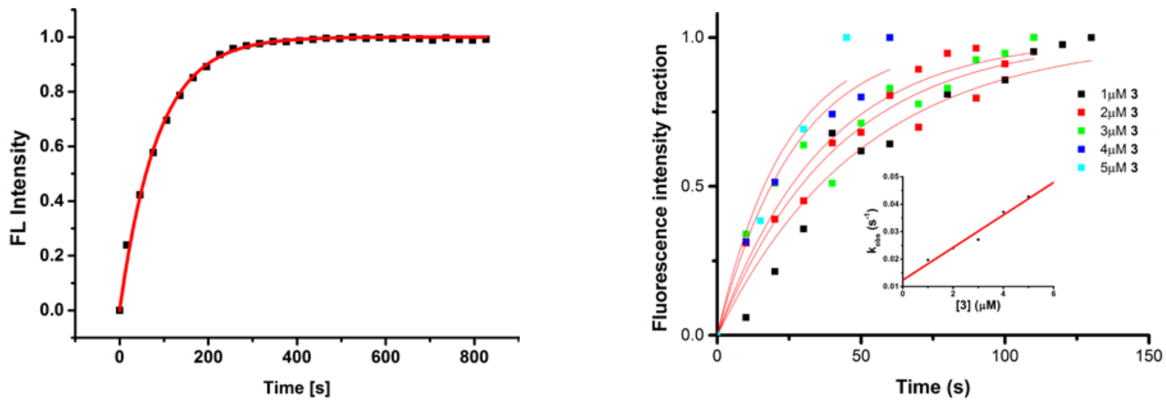

Fig. S12. Kinetic study of SNAP-tag labeling with Probe **3**. (a) Time course of fluorescence increase for the reaction of 5 μM Probe **3** with 5 μM SNAP-tag. The fluorescence intensity was recorded at 668 nm with excitation at 600 nm. (b) Fluorescence increase of Probe **3** (1−5 μM) in labeling of SNAP-tag (200 nM). The inset shows the linear relationship plot of probe concentration versus calculated k_{obs}. The second-order rate constant (k_2) for the reaction between probe **3** and SNAP-tag was determined to be about 5930 $M^{-1}s^{-1}$.

(a) **(b)**

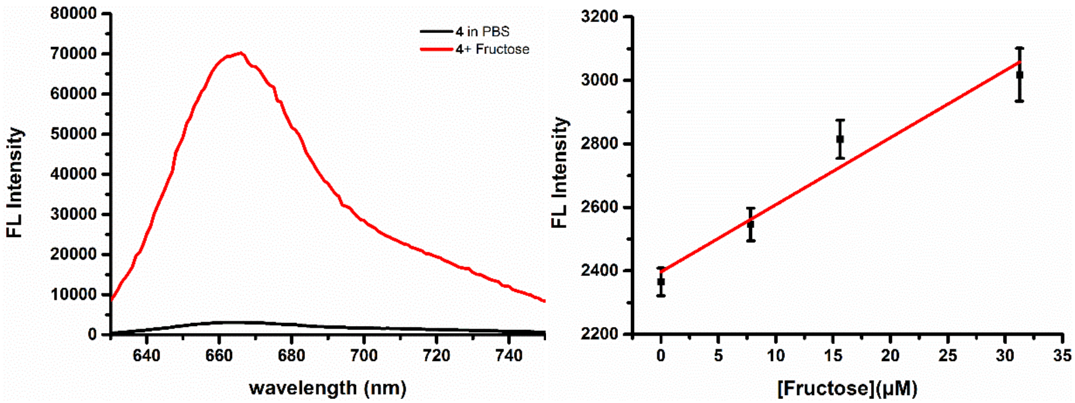

Fig. S13. (a) Fluorescence spectra of 10 μM probe **4** in the absence or presence of 5 mM fructose. (b) Linear calibration curve for the determination of fructose detection limit, which is around 10 μM fructose.

(a) (b)

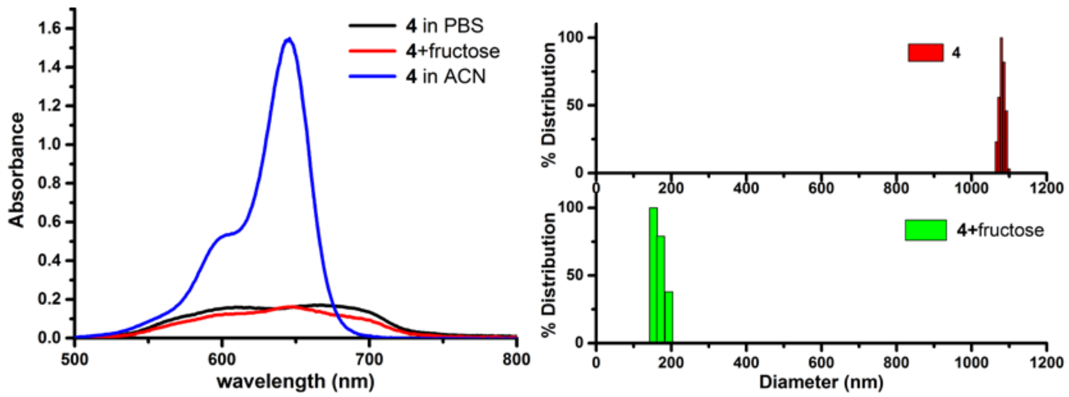

Fig. S14. (a) Absorption spectra of 10 μM probe **4** in ACN, PBS or with the addition of 5 mM fructose. (b) DLS analyses of the particle size distribution of 10 μM probe **4** and in the presence of 5 mM fructose. The mean diameter of 10 μM **4** in PBS is about 1081 nm. In the presence of 5 mM fructose, particle size was reduced to around 197 nm.

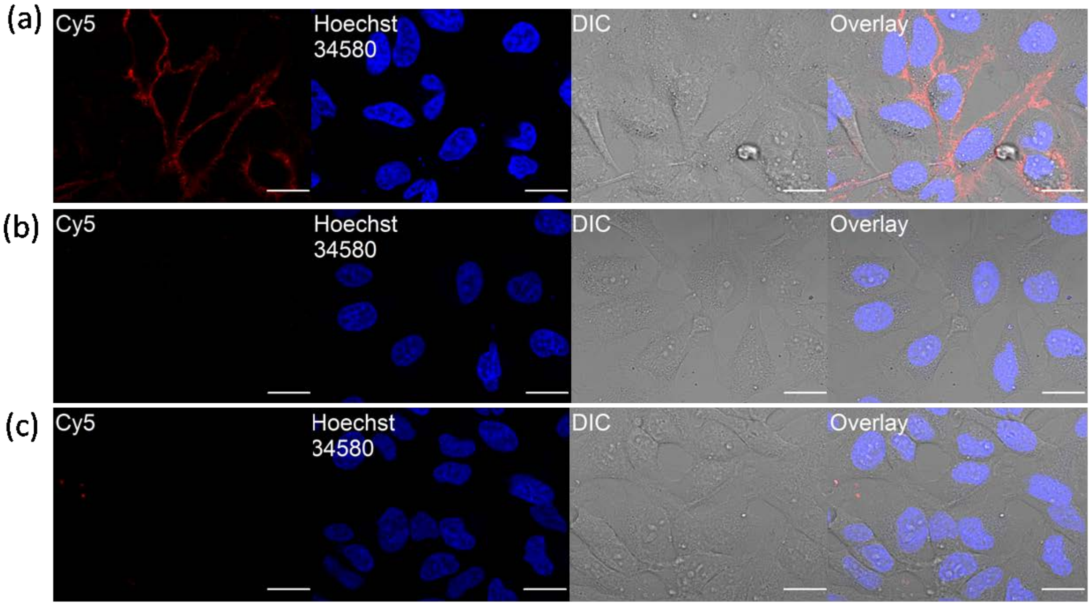

Fig. S15. No-wash live-cell imaging of HeLa cells expressing SNAP-tag on cell surface. (a) HeLa cells expressing SNAP-PDGFR proteins treated with 0.5 μM probe **3**, and (b) upon addition of 100 μM BG inhibitor. (c) Non-transfected HeLa cells treated with 0.5 μM probe **3**. All cellular images were taken on the same day with identical microscope setup. Scale bar: 20 μm.

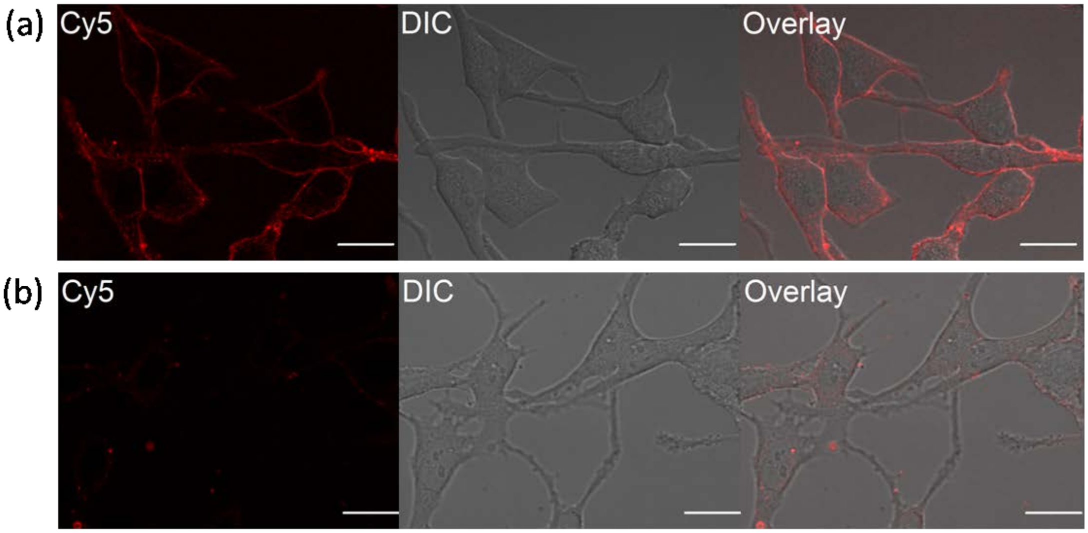

Fig. S16. HeLa cells cultured under hypoxia-mimetic condition with 200 μM DFO for 24 hours. (a) 0.5 μM probe **1** and (b) upon addition of 100 μM EZA. All cellular images were taken on the same day with identical microscope setup. Scale bar: 20 μm.

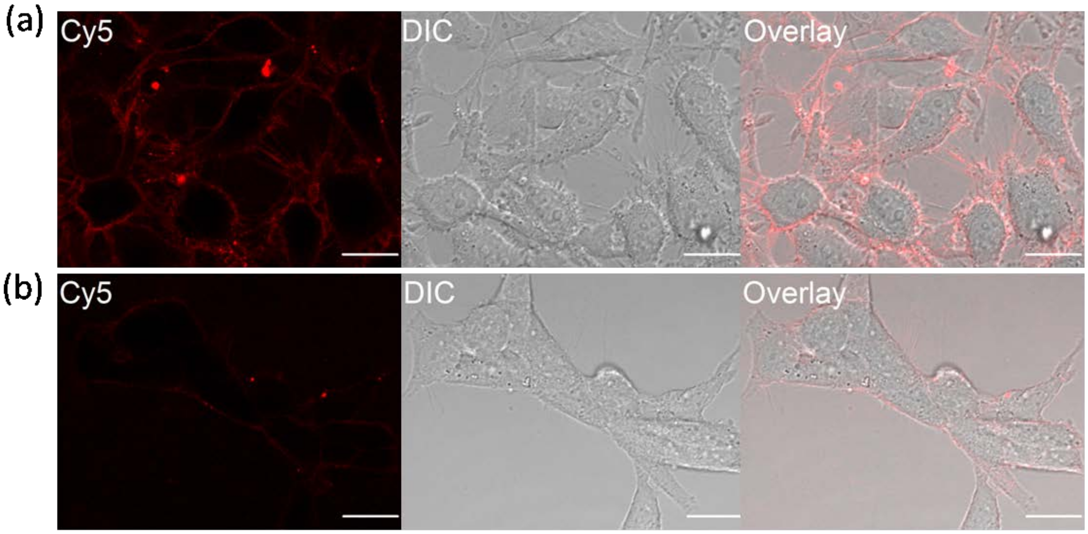

Fig. S17. No-wash imaging of hypoxia-induced (a) HeLa cells and (b) HEK293 cells. All cellular images were taken on the same day with identical microscope setup. Scale bar: 20 μm.

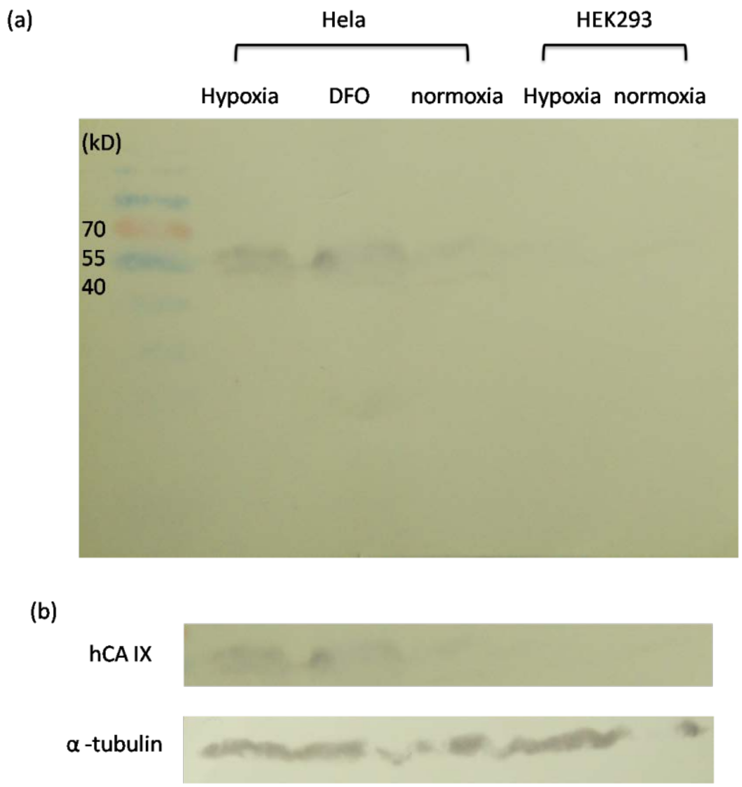

Fig. S18. Western-blot analysis of hCAIX expression levels in HeLa and HEK293 cells. (a) full view of Western-blot PVDF membrane. hCAIX was detected at around 55KDA. (b) Expanded view of PVDF membrane. α-tubulin in the cells was detected with anti-α-tubulin antibody and used as the positive control.

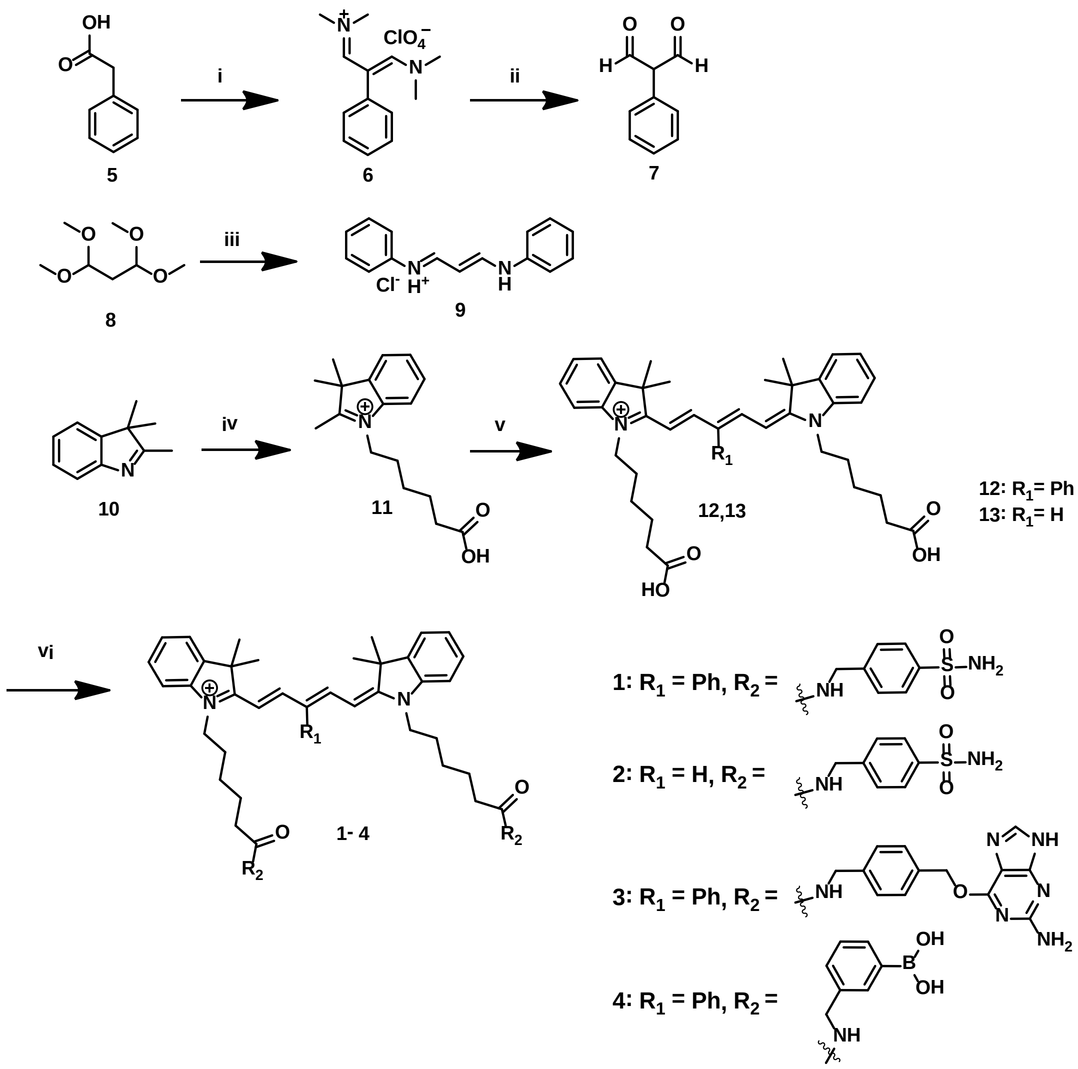

Scheme S1. Synthesis of Cy5 probe **1-4.** Reagents and conditions: (i) DMF, $POCl_3$, $NaClO_4$, 24hrs, 70%. (ii) NaOH, quantitative. (iii) HCl, aniline, 4hrs, quantitative. (iv) Bromohexanoic acid, ACN, 65%. (v) DMF, NaOAc, **7** and **11** reflux 20h, 70%; AcOH, acetic anhydride, **9** and **11** reflux 16hrs, 50%. (vi) EDC, HOBt, TEA, DMF, r.t., 8-16hrs, 50-70%.

Synthesis of compound 7

$POCl_3$ (2.1 mL, 22.5 mmol) was added dropwise to dry DMF (10 mL) at 0 °C. The mixture was stirred for one hour at room temperature. Subsequently, phenylacetic acid (1.02 g 7.5 mmol) was

added to the solution mixture. The clear solution formed was heated at 95 °C for 4 hours and then at room temperature overnight. The dark mixture was poured onto 20 g crushed ice followed by saturated $NaClO_4$solution. The resulting white crystalline was filtered and washed two times with 5 mL water to afford compound **6** in 70 % yield (1.07 g). Compound **6** was used in the next step without further purification. Compound **6** (1.07 g, 5.9 mmol) was added to 6 mL warm NaOH (1.15 g, 28.6 mmol) solution, and the mixture was heated with stirring at 90 °C until compound **6** was dissolved. The reaction mixture was cooled to room temperature and diluted with 10 mL water. The solution was acidified to pH 5 with 10% HCl solution resulting in the precipitation of the compound **7** (quantitative yield). **^{1}H NMR** (400 MHz, d_6-DMSO) δ 8.52 (s, 2H), 7.48 (d, *J* = 7.5 Hz, 2H), 7.34 (t, *J* = 7.5 Hz, 2H), 7.22 (t, *J* = 7.2 Hz, 1H) ppm; **^{13}C NMR** (101 MHz, d_6-DMSO) δ 180.05, 131.57, 129.24, 127.67, 126.64, 120.99 ppm. **HRMS (EI):** m/z calc. for $[C_9H_8O_2]^+$ 148.0524 found 148.0523 $[M]^+$.

Synthesis of compound 9

To a solution of 1,1,3,3-tetramethyl propylene oxide (1.05 ml 6 mmol) in 0.5 M HCl was added aniline (1.11 mL, 12 mmol) at room temperature. The reaction mixture was heated at 50 ℃ for four hours. The reaction mixture was filtered, washed with ether (3 X 30 mL), and the orange solid was dried under reduced pressure to give compound **11** in quantitative yield (1.5 g). R_f:0.7 (DCM: MeOH = 9: 1); **^{1}H NMR** (400 MHz, MeOD): δ 8.64 (d, *J* = 9.8 Hz, 2H), 7.47 (t, *J* = 7.9 Hz, 4H), 7.36 (d, *J* = 7.8 Hz, 4H), 7.29 (t, *J* = 7.3 Hz, 2H), 6.23 (t, *J* = 11.6 Hz, 1H) ppm; **^{13}C NMR** (126 MHz, MeOD) δ 159.79, 139.81, 131.14, 127.70, 118.76, 99.37 ppm; **HRMS** (ESI): m/z calc. for $[C_{15}H_{15}N_2]^+$ 223.1230 found 223.1230 $[M]^+$.

Synthesis of compound 11

To a stirred solution of 2,3,3-trimethyl-3H-indole (1 g, 6.28 mmol) in acetonitrile (5 mL) was added 6-bromohexanoic acid (1.2 g, 6.28 mmol) at room temperature. The reaction flask was fitted with a reflux condenser and heated at reflux for 24 hours. Subsequently, the heating bath was removed and the reaction mixture was allowed to cool to -20°C. The precipitates was filtered and dried under vacuo to provide compound **11** as a purple solid in 65% yield (1.3 g). R_f: 0.1 (DCM: MeOH = 9:1); ^{1}**H NMR** (400 MHz, d_6-DMSO): δ 8.00 (dd, *J* = 6.0, 2.9 Hz, 1H), 7.85 (dd, *J* = 6.0, 2.7 Hz, 1H), 7.65 – 7.49 (m, 2H), 4.47 (t, *J* = 7.7 Hz, 2H), 2.87 (s, 3H), 2.21 (t, *J* = 7.2 Hz, 2H), 1.95 – 1.74 (m, 2H), 1.53 (s, 8H), 1.47 – 1.37 (m, 2H) ppm; ^{13}**C NMR** (101 MHz, d_6-DMSO): δ 196.50, 174.28, 141.85, 141.02, 129.34, 128.90, 123.52, 115.52, 54.15, 47.49, 33.37, 26.94, 25.38, 24.01, 22.00, 14.22 ppm; **HRMS** (ESI): m/z calc. for $[C_{17}H_{24}NO_2]^+$ 274.1802 found 274.1800 $[M]^+$.

Synthesis of compound 12

Sodium acetate (31 mg, 0.39 mmol) and **7** (52 mg, 0.35 mmol) were added to a solution of **11** (300 mg, 0.88 mmol) in dry ethanol (15 mL) at room temperature. The reaction flask was fitted with a reflux condenser and heated at reflux for 20 hours. The solvent was removed and the residue was purified by flash column chromatography (MeOH/DCM) to afford compound **12** as a blue solid in 70% yield (160 mg). ^{1}**H NMR** (400 MHz, MeOD) δ 8.33 (d, *J* = 14.0 Hz, 2H), 7.62 (t, *J* = 7.6 Hz, 2H), 7.57 – 7.43 (m, 3H), 7.37 (t, *J* = 7.7 Hz, 2H), 7.32 – 7.18 (m, 6H), 5.73 (d, *J* = 14.0 Hz, 2H), 3.73 (t, *J* = 7.3 Hz, 4H), 2.17 (t, *J* = 7.3 Hz, 4H), 1.76 (s, 12H), 1.66 – 1.53 (m, 4H), 1.53 – 1.40 (m, 4H), 1.31 – 1.11 (m, 4H) ppm; ^{13}**C NMR** (101 MHz, MeOD) δ 181.88, 174.54, 154.08, 143.43, 142.62, 137.13, 136.86, 131.28, 130.58 , 129.73, 129.40, 126.37, 123.39, 112.05, 102.89, 50.50, 44.97, 38.54, 28.07, 27.9, 27.82, 27.05 ppm; **HRMS** (ESI): m/z calc. for

$[C_{43}H_{51}N_2O_4]^+$ 659.3843 found 659.3878 $[M]^+$.

Synthesis of compound 13

To compound **9** (254 mg 0.717 mmol) in a mixture of 10 mL $(Ac)_2O$/AcOH (1:1) was added compound **11** (195 mg 0.79 mmol) at room temperature. The reaction was heated at reflux for 16 hours. The solvent was removed and the residue was purified by flash-column chromatography (MeOH/DCM) to afford compound **13** as a blue solid in 50% yield (209 mg). **^{1}H NMR** (400 MHz, d_6-DMSO) δ 8.51 (d, *J* = 12.7 Hz, 1H), 8.40 – 8.25 (m, 2H), 7.58 (d, *J* = 7.4 Hz, 2H), 7.46 – 7.28 (m, 2H), 7.26 – 7.12 (m, 2H), 7.07 – 6.88 (m, 1H), 6.67 – 6.52 (m, 1H), 6.23 (d, *J* = 13.7 Hz, 2H), 4.03 (s, 4H), 2.05 (s, 4H), 1.62 (s, 12H), 1.51 – 1.06 (m, 8H) ppm; **^{13}C NMR** (126 MHz, d_6-DMSO) δ 172.55, 153.93, 141.88, 141.14, 128.49, 126.08, 125.98, 124.76, 122.54, 111.07, 103.28, 48.93, 43.29, 36.11, 27.17, 26.64, 25.84, 24.93 ppm; **HRMS** (ESI): m/z calc. for $[C_{37}H_{47}N_2O_4]^+$ 583.3530 found 583.3532 $[M]^+$.

Synthesis of compound 1

To a reaction flask containing compound **6** (15 mg, 0.02 mmol), HOBt.H_2O (8 mg, 0.06 mmol), and EDC.HCl (12 mg, 0.06 mmol, 3 equiv) was added 2 mL DMF at room temperature. After 10 minutes, triethylamine (28 uL) and 4-aminomethylbenzenesulfonamide hydrochloride (13 mg, 0.06 mmol) were added respectively and stirred overnight at room temperature. The solvent was removed and the crude mixture was purified by reversed-phase preparative HPLC to give product **1** as a blue solid in 65% yield (13 mg). **^{1}H NMR** (400 MHz, MeOD) δ 8.34 (d, *J* = 14.0 Hz, 2H), 7.83 (d, *J* = 8.3 Hz, 4H), 7.56 (t, *J* = 7.7 Hz, 2H), 7.49 (d, *J* = 7.4 Hz, 2H), 7.42 (d, *J* = 8.2 Hz, 5H), 7.37 (d, *J* = 7.8 Hz, 2H), 7.31 – 7.18 (m, 6H), 5.73 (d, *J* = 13.9 Hz, 2H), 4.41 (s, 4H), 3.74 (t, *J* = 7.4 Hz, 4H), 2.20 (t, *J* = 7.3 Hz, 4H), 1.76 (s, 12H), 1.62 – 1.48 (m, 8H), 1.25 –

1.16 (m, 4H) ppm; **^{13}C NMR** (101 MHz, MeOD) δ 175.66, 174.64, 154.09, 144.85, 143.81, 143.37, 142.63, 137.27, 136.79, 131.34, 130.54, 129.78, 129.34, 128.93, 127.40, 126.48, 123.47, 112.02, 102.90, 50.54, 44.82, 43.59, 36.62, 27.91, 27.83, 27.43, 26.32 ppm; **HRMS** (ESI): m/z calc. for $[C_{57}H_{67}N_6O_6S_2]^+$ 995.4558 found 995.4559 $[M]^+$.

Synthesis of compound 2

Based on the synthetic procedure of compound **1,** compound **2** was obtained as a blue solid in 50 % yield (9 mg). **^{1}H NMR** (500 MHz, d_6-DMSO) δ 8.40 (t, *J* = 6.0 Hz, 2H), 8.37 – 8.28 (m, 2H), 7.74 (d, *J* = 8.3 Hz, 3H), 7.61 (d, *J* = 7.4 Hz, 2H), 7.38 (t, *J* = 7.1 Hz, 5H), 7.31 (s, 2H), 7.27 – 7.21 (m, 2H), 6.58 (t, *J* = 12.3 Hz, 1H), 6.29 (d, *J* = 13.9 Hz, 2H), 4.28 (d, *J* = 5.9 Hz, 4H), 4.07 (d, *J* = 7.2 Hz, 4H), 2.14 (t, *J* = 7.3 Hz, 4H), 1.67 (s, 12H), 1.60 – 1.51 (m, 4H), 1.36 (d, *J* = 7.0 Hz, 4H), 1.23 (d, *J* = 9.9 Hz, 4H) ppm; **^{13}C NMR** (126 MHz, d_6-DMSO) δ 172.59, 172.09, 154.03, 143.78, 142.55, 142.00, 141.09, 128.45, 127.42, 125.66, 124.70, 122.46, 111.10, 103.16, 48.90, 43.30, 41.64, 35.04, 27.16, 26.73, 25.80, 24.90 ppm; **HRMS** (ESI): m/z calc. for $[C_{51}H_{63}N_6O_6S_2]^+$ 919.4245 found 919.4199 $[M]^+$.

Synthesis of compound 3

Based on the synthetic procedure of compound **1,** compound **3** was obtained as a blue solid in 70 % yield (16 mg). **^{1}H NMR** (400 MHz, MeOD) δ 8.35 (d, *J* = 14.0 Hz, 2H), 8.27 (s, 2H), 7.58 (t, *J* = 7.5 Hz, 2H), 7.47 (t, *J* = 6.3 Hz, 7H), 7.38 – 7.27 (m, 8H), 7.24 (t, *J* = 7.2 Hz, 2H), 7.17 (d, *J* = 8.0 Hz, 2H), 5.73 (d, *J* = 13.9 Hz, 2H), 5.60 (s, 4H), 4.35 (s, 4H), 3.72 (t, *J* = 7.3 Hz, 4H), 2.18 (t, *J* = 7.3 Hz, 4H), 1.76 (s, 12H), 1.61 – 1.46 (m, 8H), 1.21 (dd, *J* = 14.9, 8.1 Hz, 4H) ppm; **^{13}C NMR** (101 MHz, MEOD) δ 175.52, 174.64, 161.10, 158.44, 154.14, 153.75, 143.34, 142.63, 140.95, 137.27, 136.83, 135.47, 131.36, 130.53, 130.21, 129.74, 129.33, 128.84, 126.44, 123.45,

111.97, 102.88, 70.69, 50.54, 44.82, 43.78, 36.64, 27.90, 27.80, 27.43, 26.36 ppm; **HRMS** (ESI): m/z calc. for $[C_{69}H_{75}N_{14}O_4]^+$ 1163.6090 found 1163.6099 $[M]^+$.

Synthesis of compound 4

Based on the synthetic procedure of compound **1,** compound **4** was obtained as a blue solid in 70 % yield (13 mg). **^{1}H NMR** (500 MHz, MeOD) δ 8.42 – 8.29 (m, 3H), 7.56 (t, *J* = 7.6 Hz, 3H), 7.49 (d, *J* = 7.4 Hz, 3H), 7.44 (s, 2H), 7.37 (t, *J* = 7.7 Hz, 2H), 7.27 (dt, *J* = 15.5, 7.6 Hz, 8H), 7.20 (d, *J* = 8.0 Hz, 2H), 5.72 (d, *J* = 14.0 Hz, 2H), 4.34 (s, 4H), 3.72 (t, *J* = 7.3 Hz, 4H), 2.17 (t, *J* = 7.3 Hz, 4H), 1.76 (s, 12H), 1.62 – 1.46 (m, 8H), 1.25 – 1.15 (m, 4H) ppm; **^{13}C NMR** (126 MHz, MeOD) δ 175.45, 154.12, 143.36, 142.63, 134.27, 133.81, 131.32, 130.56, 129.78, 129.37, 128.81, 126.45, 123.46, 112.02, 102.89, 49.85, 44.79 , 44.26 , 36.65 , 27.87, 27.79, 27.35 , 26.36 ppm; **HRMS** (ESI): m/z calc. for $[C_{57}H_{67}B_2N_4O_6+H]^+$ 926.5319 found 926.5327 $[M+H]^+$.

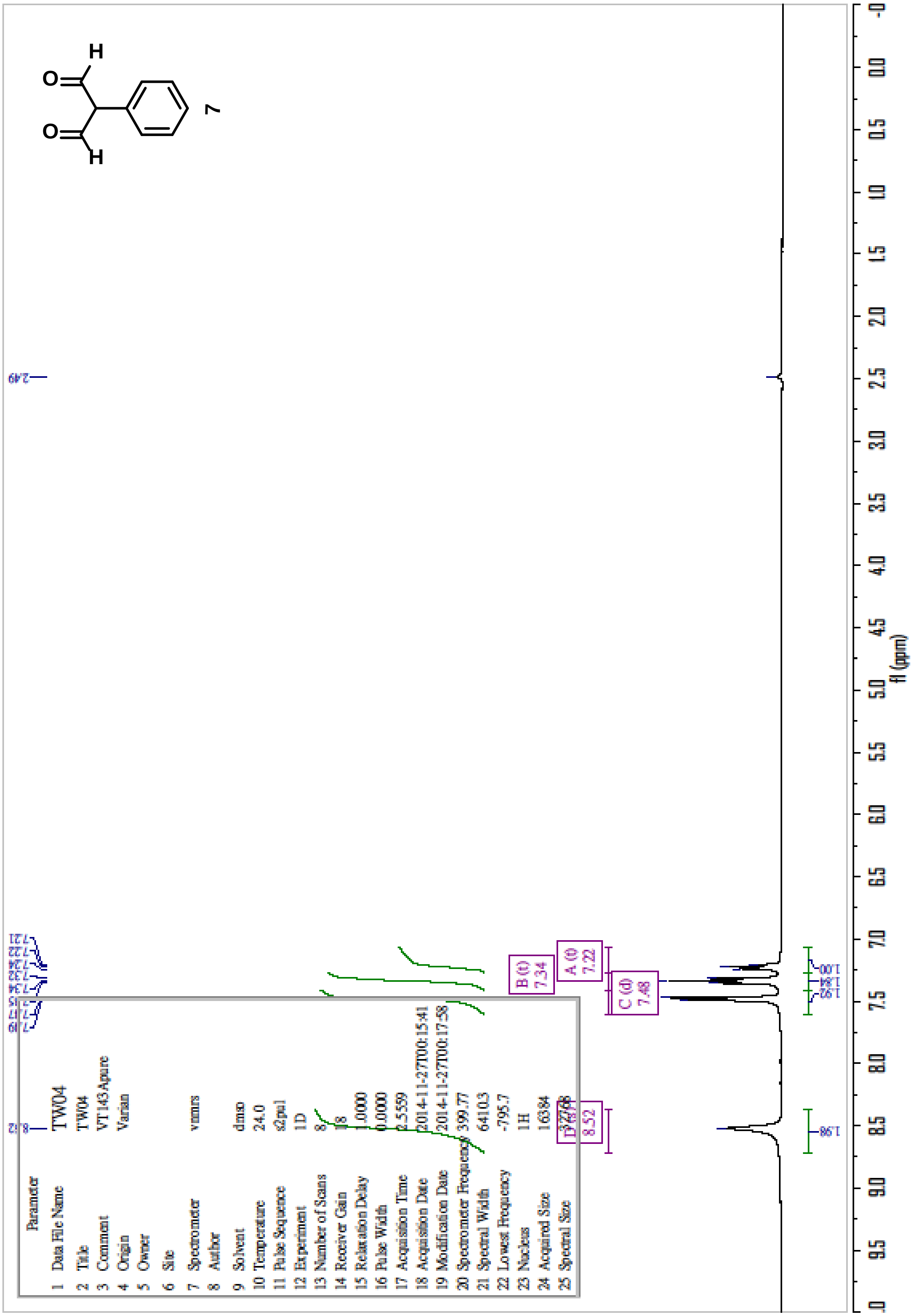

7
f1 (ppm)
Parameter
1 Data File Name TW04
2 Title TW04
3 Comment VT143Apure
4 Origin Varian
5 Owner
6 Site
7 Spectrometer vnmrs
8 Author
9 Solvent dmso
10 Temperature 24.0
11 Pulse Sequence s2pul
12 Experiment 1D
13 Number of Scans 8
14 Receiver Gain 18
15 Relaxation Delay 1.0000
16 Pulse Width 0.0000
17 Acquisition Time 2.5559
18 Acquisition Date 2014-11-27T00:15:41
19 Modification Date 2014-11-27T00:17:58
20 Spectrometer Frequency 399.77
21 Spectral Width 6410.3
22 Lowest Frequency -795.7
23 Nucleus 1H
24 Acquired Size 16384
25 Spectral Size 32768
A (t) 7.22
B (t) 7.34
C (d) 7.48
D (s) 8.52

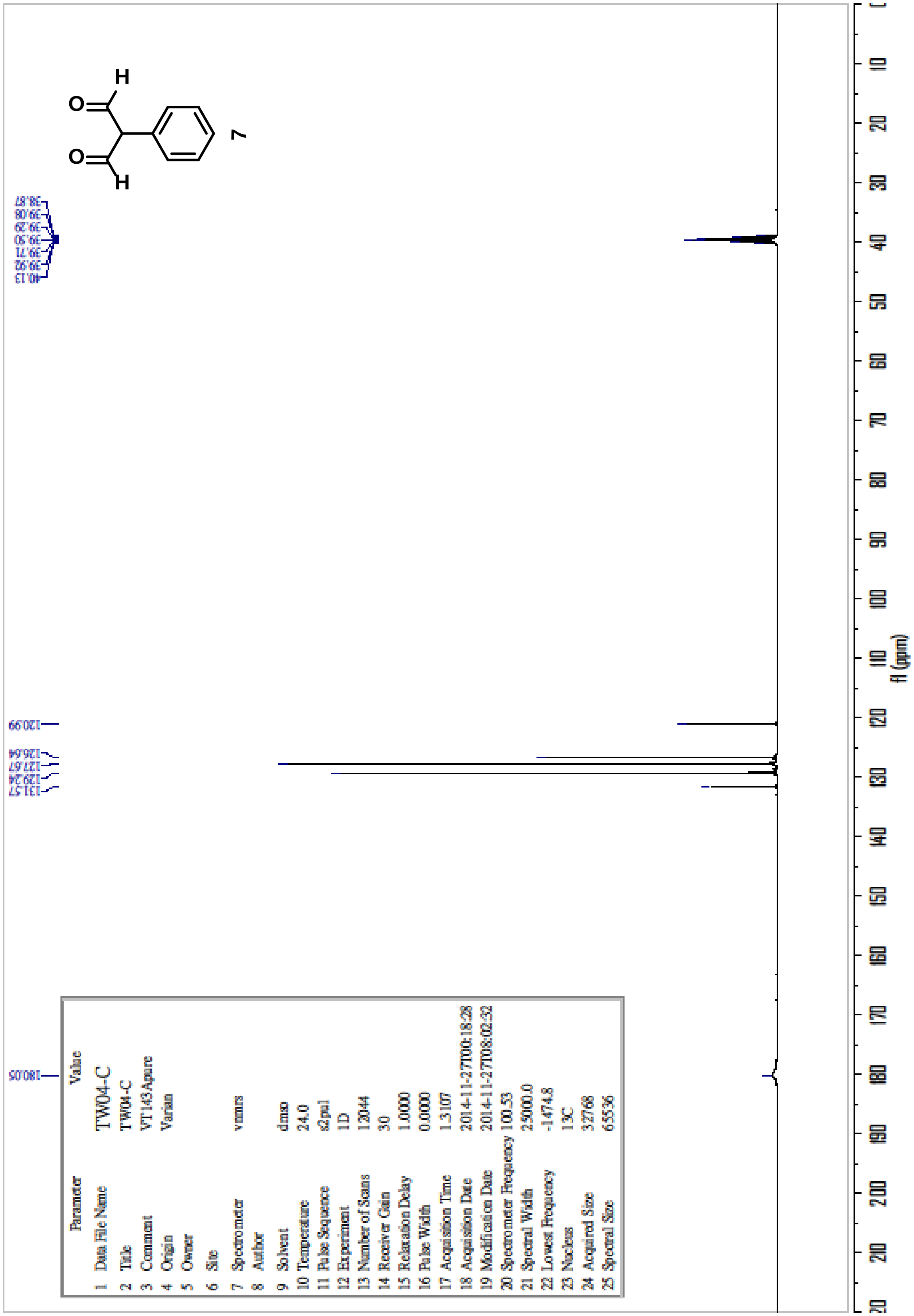

7
38.87
39.08
39.29
39.50
39.71
39.92
40.13
120.99
126.64
127.67
129.24
131.57
180.05
Parameter Value
1 Data File Name TW04-C
2 Title TW04-C
3 Comment VT143Apure
4 Origin Varian
5 Owner
6 Site
7 Spectrometer vnmrs
8 Author
9 Solvent dmso
10 Temperature 24.0
11 Pulse Sequence s2pul
12 Experiment 1D
13 Number of Scans 12044
14 Receiver Gain 30
15 Relaxation Delay 1.0000
16 Pulse Width 0.0000
17 Acquisition Time 1.3107
18 Acquisition Date 2014-11-27T00:18:28
19 Modification Date 2014-11-27T08:02:32
20 Spectrometer Frequency 100.53
21 Spectral Width 25000.0
22 Lowest Frequency -1474.8
23 Nucleus 13C
24 Acquired Size 32768
25 Spectral Size 65536
f1 (ppm)

	Parameter	Value
1	Title	PI66
2	Origin	Varian
3	Owner	
4	Site	
5	Spectrometer	mercury
6	Author	aer7917
7	Solvent	DMSO
8	Temperature	29.0
9	Pulse Sequence	s2pul
10	Experiment	1D
11	Number of Scans	16
12	Receiver Gain	20
13	Relaxation Delay	1.0000
14	Pulse Width	0.0000
15	Acquisition Time	1.9950
16	Acquisition Date	2014-05-16T12:11:27
17	Modification Date	2014-05-16T12:17:00
18	Spectrometer Frequency	400.44
19	Spectral Width	6006.0
20	Lowest Frequency	-982.8
21	Nucleus	1H
22	Acquired Size	11982
23	Spectral Size	32768

^{1}H NMR (400 MHz, DMSO) δ 8.00 (dd, J = 6.0, 2.9 Hz, 1H), 7.85 (dd, J = 6.0, 2.7 Hz, 1H), 7.65 – 7.49 (m, 2H), 4.47 (t, J = 7.7 Hz, 2H), 2.87 (s, 3H), 2.21 (t, J = 7.2 Hz, 2H), 1.95 – 1.74 (m, 2H), 1.53 (s, 8H), 1.47 – 1.37 (m, 2H).

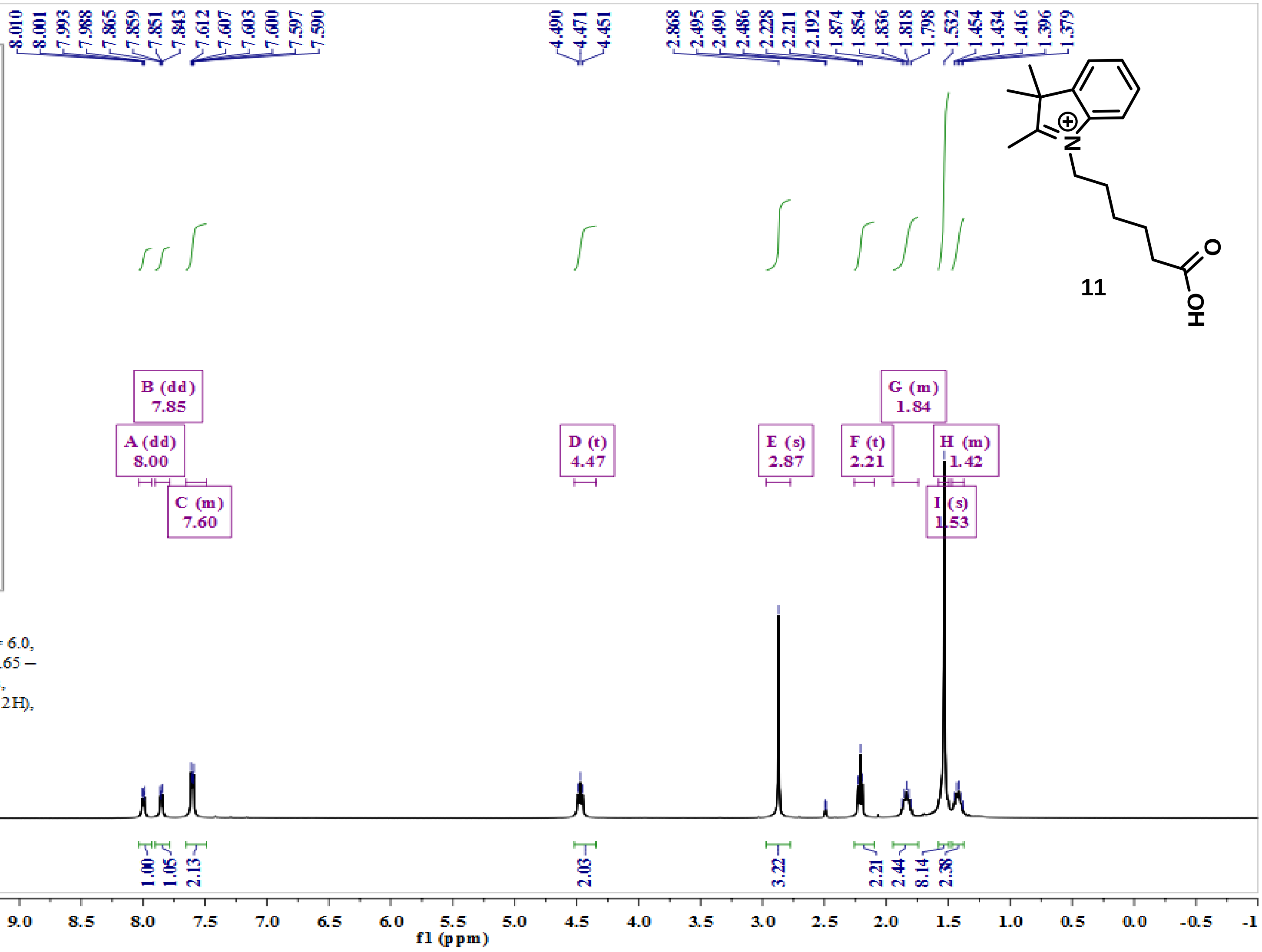

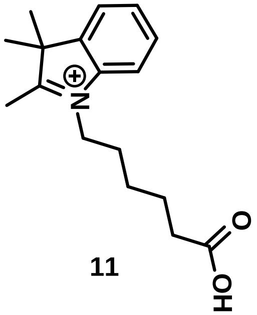

11

Parameter	Value
1 Title	PI66-carbon
2 Origin	Varian
3 Owner	
4 Site	
5 Spectrometer	mercury
6 Author	aer7917
7 Solvent	DMSO
8 Temperature	29.0
9 Pulse Sequence	s2pul
10 Experiment	1D
11 Number of Scans	484
12 Receiver Gain	36
13 Relaxation Delay	0.0000
14 Pulse Width	0.0000
15 Acquisition Time	1.1994
16 Acquisition Date	2014-05-16T12:12:53
17 Modification Date	2014-05-16T12:31:00
18 Spectrometer Frequency	100.70
19 Spectral Width	25000.0
20 Lowest Frequency	-3040.3
21 Nucleus	13C
22 Acquired Size	29984
23 Spectral Size	65536

^{13}C NMR (101 MHz, DMSO) δ 196.50, 174.28, 141.85, 141.02, 129.34, 128.90, 123.52, 115.52, 54.15, 47.49, 33.37, 26.94, 25.38, 24.01, 22.00, 14.22.

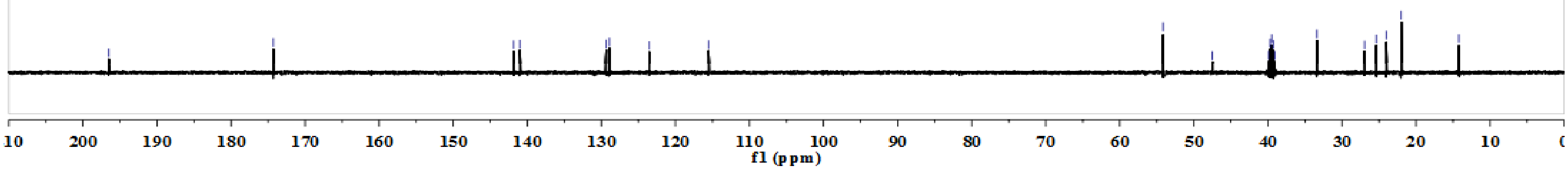

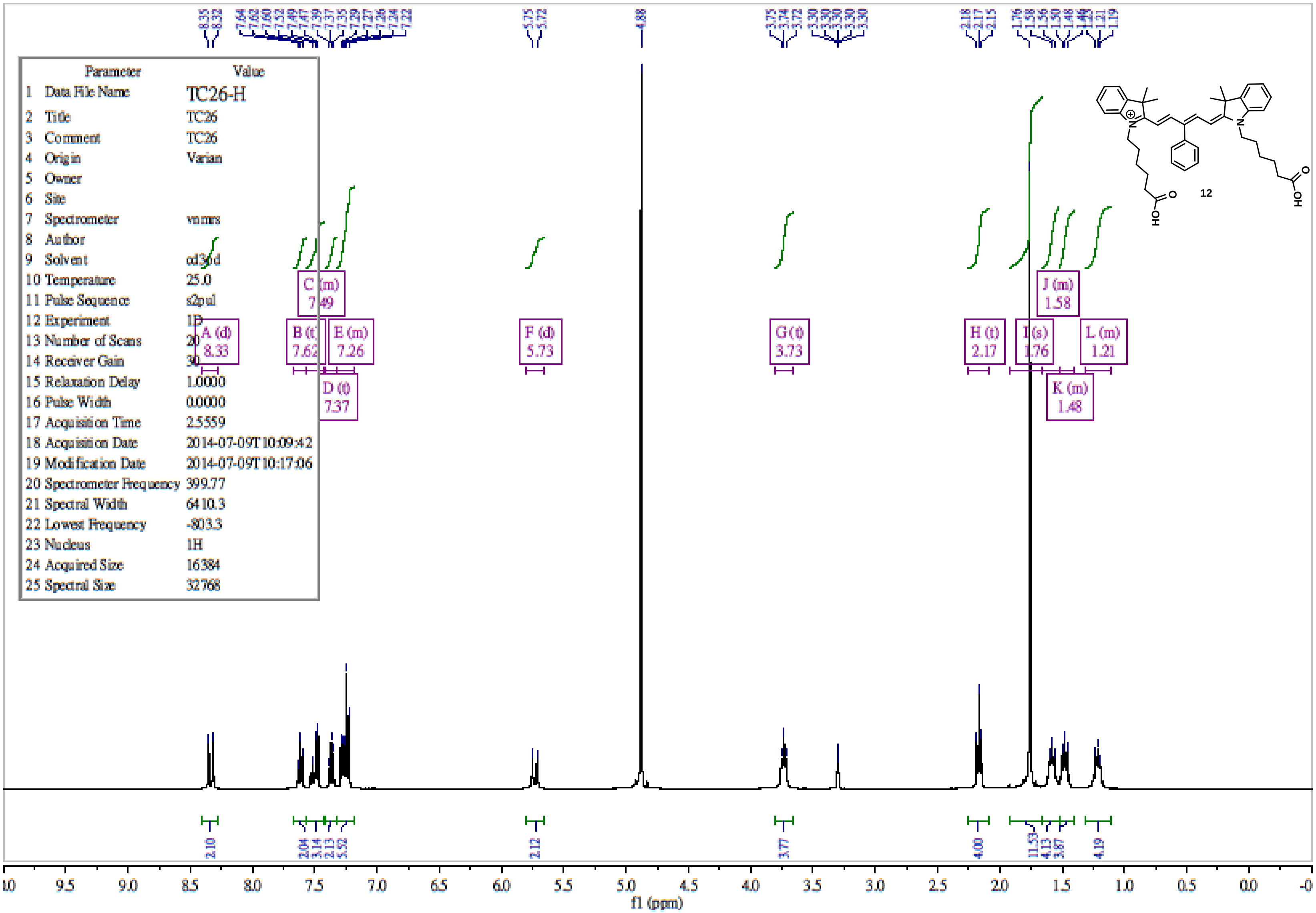
Parameter Value
1 Data File Name TC26-H
2 Title TC26
3 Comment TC26
4 Origin Varian
5 Owner
6 Site
7 Spectrometer vnmrs
8 Author
9 Solvent cd3od
10 Temperature 25.0
11 Pulse Sequence s2pul
12 Experiment 1D
13 Number of Scans 20
14 Receiver Gain 30
15 Relaxation Delay 1.0000
16 Pulse Width 0.0000
17 Acquisition Time 2.5559
18 Acquisition Date 2014-07-09T10:09:42
19 Modification Date 2014-07-09T10:17:06
20 Spectrometer Frequency 399.77
21 Spectral Width 6410.3
22 Lowest Frequency -803.3
23 Nucleus 1H
24 Acquired Size 16384
25 Spectral Size 32768
A (d) 8.33
B (t) 7.62
C (m) 7.49
D (t) 7.37
E (m) 7.26
F (d) 5.73
G (t) 3.73
H (t) 2.17
I (s) 1.76
J (m) 1.58
K (m) 1.48
L (m) 1.21
12
f1 (ppm)

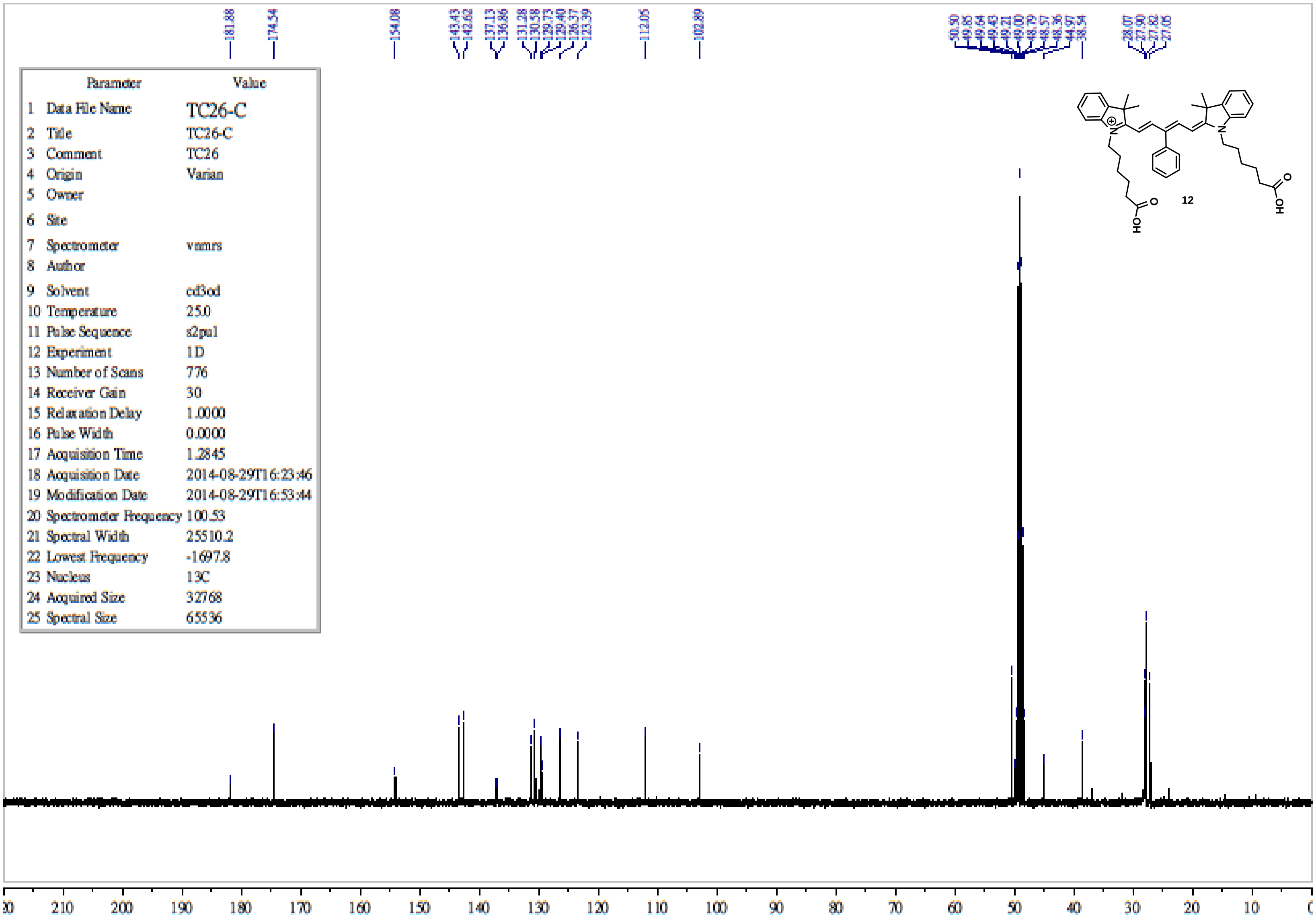

Parameter	Value
1 Data File Name	TC26-C
2 Title	TC26-C
3 Comment	TC26
4 Origin	Varian
5 Owner	
6 Site	
7 Spectrometer	vnmrs
8 Author	
9 Solvent	cd3od
10 Temperature	25.0
11 Pulse Sequence	s2pul
12 Experiment	1D
13 Number of Scans	776
14 Receiver Gain	30
15 Relaxation Delay	1.0000
16 Pulse Width	0.0000
17 Acquisition Time	1.2845
18 Acquisition Date	2014-08-29T16:23:46
19 Modification Date	2014-08-29T16:53:44
20 Spectrometer Frequency	100.53
21 Spectral Width	25510.2
22 Lowest Frequency	-1697.8
23 Nucleus	13C
24 Acquired Size	32768
25 Spectral Size	65536

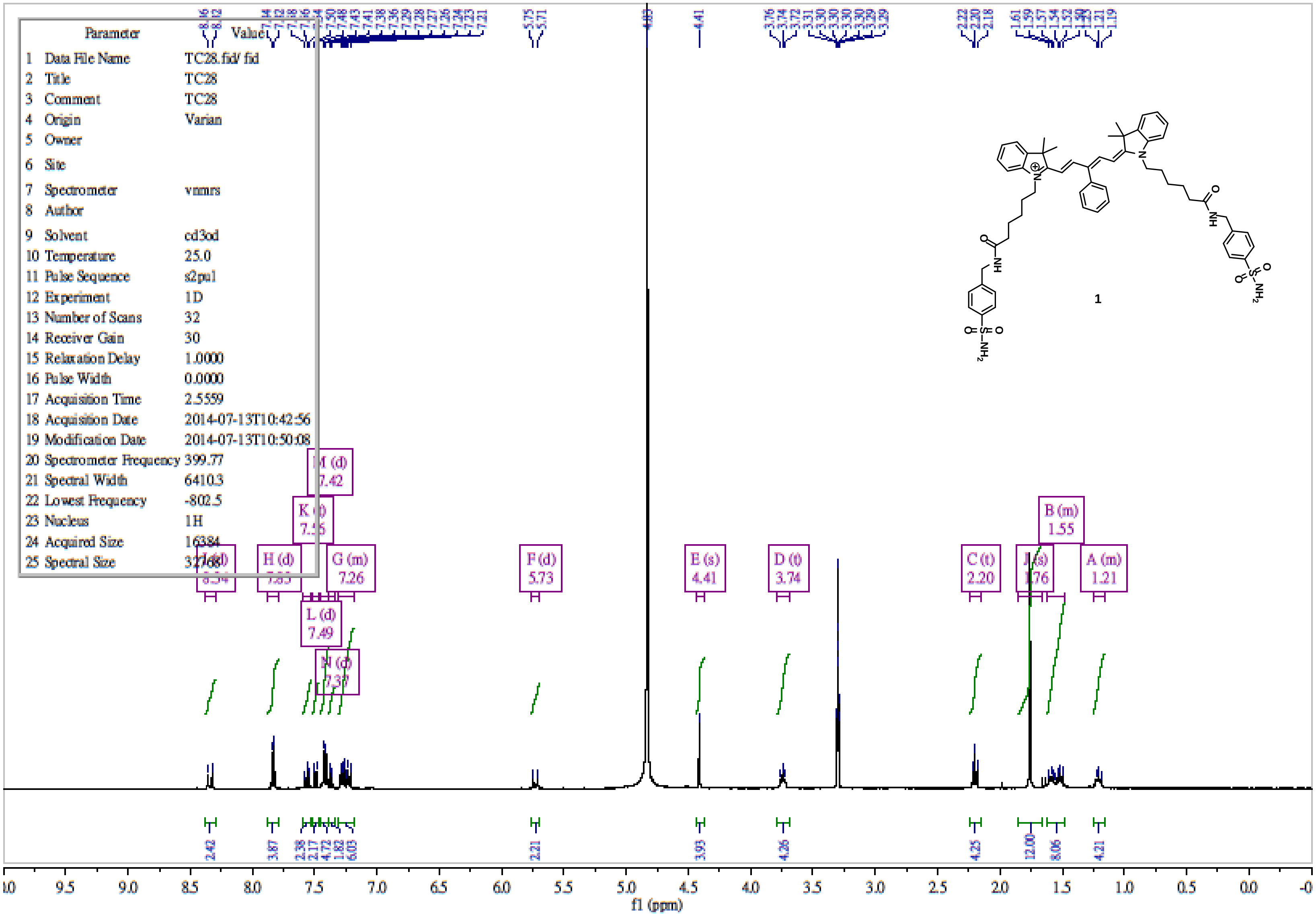
Parameter Value
1 Data File Name TC28.fid/ fid
2 Title TC28
3 Comment TC28
4 Origin Varian
5 Owner
6 Site
7 Spectrometer vnmrs
8 Author
9 Solvent cd3od
10 Temperature 25.0
11 Pulse Sequence s2pul
12 Experiment 1D
13 Number of Scans 32
14 Receiver Gain 30
15 Relaxation Delay 1.0000
16 Pulse Width 0.0000
17 Acquisition Time 2.5559
18 Acquisition Date 2014-07-13T10:42:56
19 Modification Date 2014-07-13T10:50:08
20 Spectrometer Frequency 399.77
21 Spectral Width 6410.3
22 Lowest Frequency -802.5
23 Nucleus 1H
24 Acquired Size 16384
1
F (d) 5.73
E (s) 4.41
D (t) 3.74
C (t) 2.20
B (m) 1.55
A (m) 1.21
G (m) 7.26
L (d) 7.49
f1 (ppm)

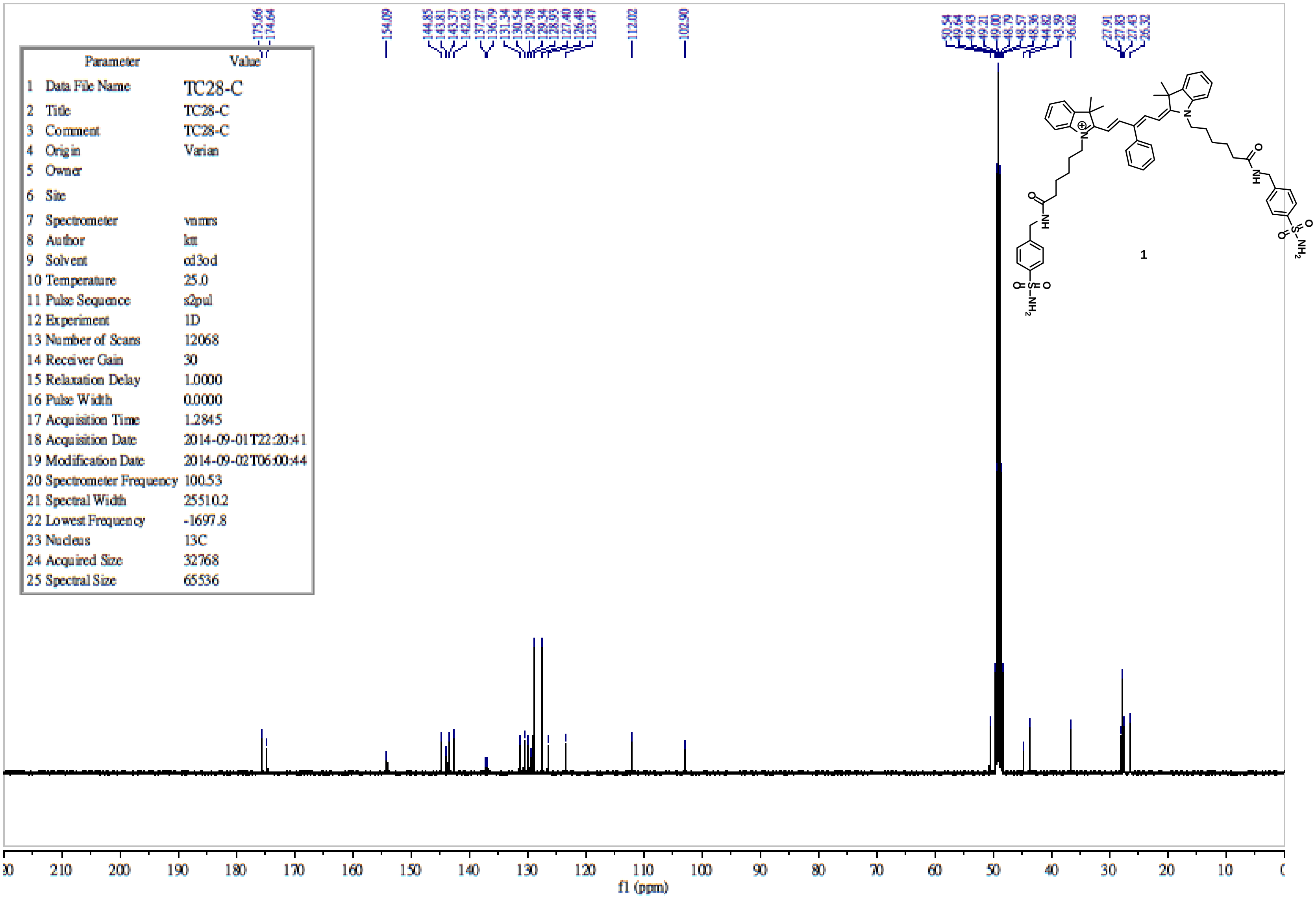
Parameter Value
1 Data File Name TC28-C
2 Title TC28-C
3 Comment TC28-C
4 Origin Varian
5 Owner
6 Site
7 Spectrometer vnmrs
8 Author ktt
9 Solvent cd3od
10 Temperature 25.0
11 Pulse Sequence s2pul
12 Experiment 1D
13 Number of Scans 12068
14 Receiver Gain 30
15 Relaxation Delay 1.0000
16 Pulse Width 0.0000
17 Acquisition Time 1.2845
18 Acquisition Date 2014-09-01T22:20:41
19 Modification Date 2014-09-02T06:00:44
20 Spectrometer Frequency 100.53
21 Spectral Width 25510.2
22 Lowest Frequency -1697.8
23 Nucleus 13C
24 Acquired Size 32768
25 Spectral Size 65536
175.66
174.64
154.09
144.85
143.81
143.37
142.63
137.27
136.79
131.34
130.54
129.78
129.34
128.93
127.40
126.48
123.47
112.02
102.90
50.54
49.64
49.43
49.21
49.00
48.79
48.57
48.36
44.82
43.59
36.62
27.91
27.83
27.43
26.32
1
f1 (ppm)

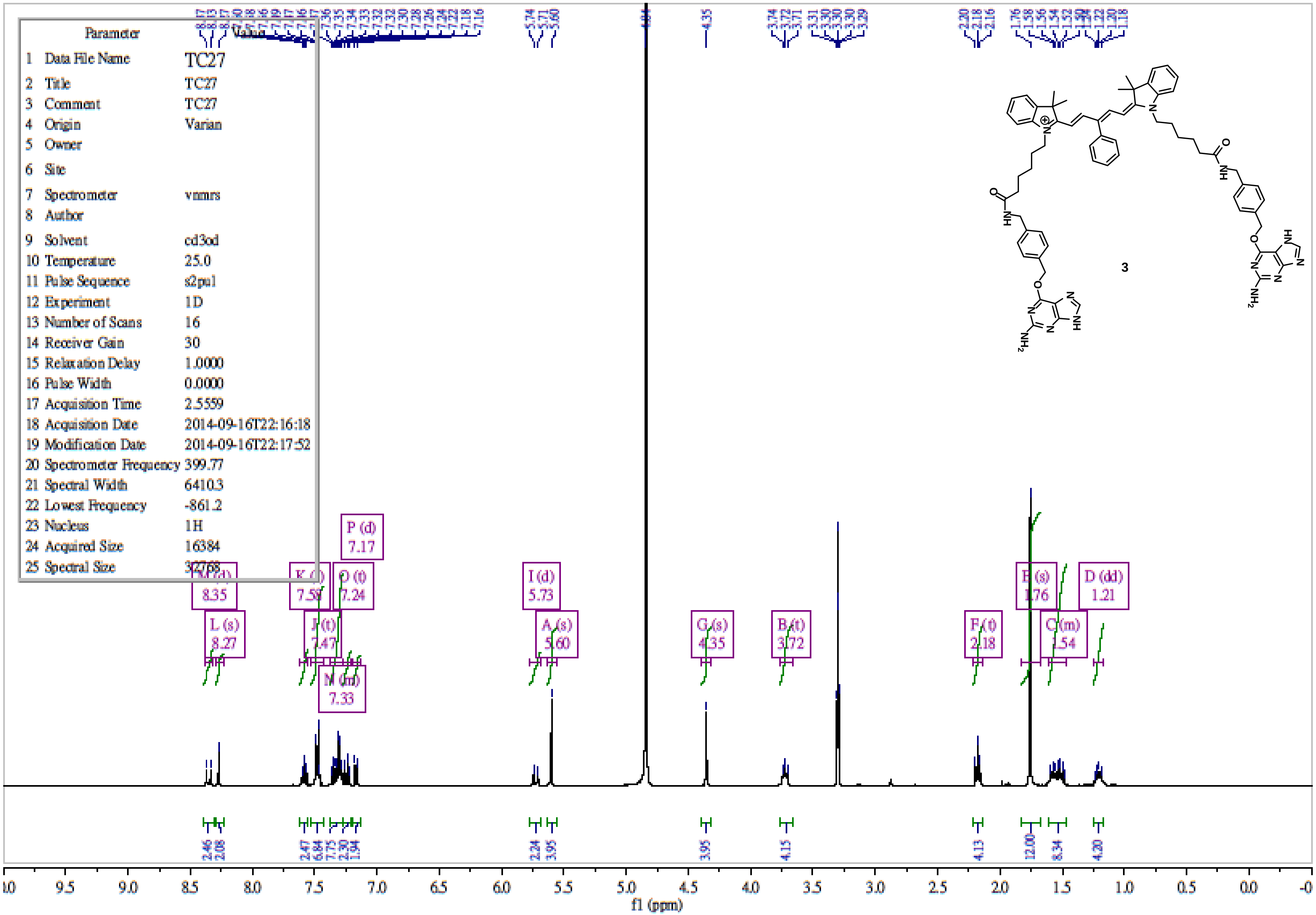

	Parameter	Value
1	Data File Name	TC27
2	Title	TC27
3	Comment	TC27
4	Origin	Varian
5	Owner	
6	Site	
7	Spectrometer	vnmrs
8	Author	
9	Solvent	cd3od
10	Temperature	25.0
11	Pulse Sequence	s2pul
12	Experiment	1D
13	Number of Scans	16
14	Receiver Gain	30
15	Relaxation Delay	1.0000
16	Pulse Width	0.0000
17	Acquisition Time	2.5559
18	Acquisition Date	2014-09-16T22:16:18
19	Modification Date	2014-09-16T22:17:52
20	Spectrometer Frequency	399.77
21	Spectral Width	6410.3
22	Lowest Frequency	-861.2
23	Nucleus	1H
24	Acquired Size	16384
25	Spectral Size	32768

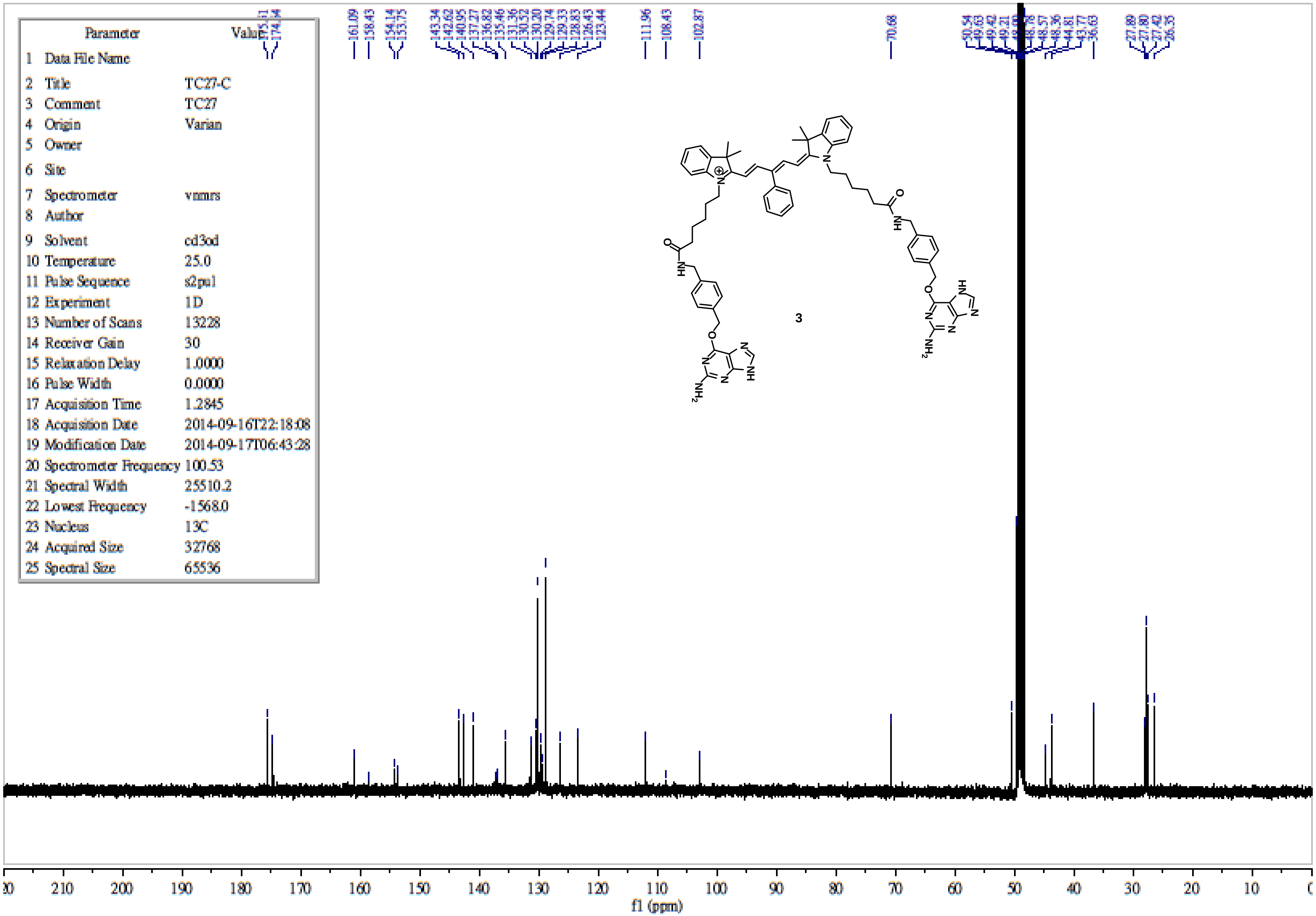

Parameter Value
1 Data File Name
2 Title TC27-C
3 Comment TC27
4 Origin Varian
5 Owner
6 Site
7 Spectrometer vnmrs
8 Author
9 Solvent cd3od
10 Temperature 25.0
11 Pulse Sequence s2pul
12 Experiment 1D
13 Number of Scans 13228
14 Receiver Gain 30
15 Relaxation Delay 1.0000
16 Pulse Width 0.0000
17 Acquisition Time 1.2845
18 Acquisition Date 2014-09-16T22:18:08
19 Modification Date 2014-09-17T06:43:28
20 Spectrometer Frequency 100.53
21 Spectral Width 25510.2
22 Lowest Frequency -1568.0
23 Nucleus 13C
24 Acquired Size 32768
25 Spectral Size 65536
175.11
174.34
161.09
158.43
154.14
153.75
143.34
142.62
140.95
137.27
136.82
135.46
131.36
130.52
130.20
129.74
129.33
128.83
126.43
123.44
111.96
108.43
102.87
70.68
50.54
49.63
49.42
49.21
48.78
48.57
48.36
44.81
43.77
36.63
27.89
27.80
27.42
26.35
3
f1 (ppm)

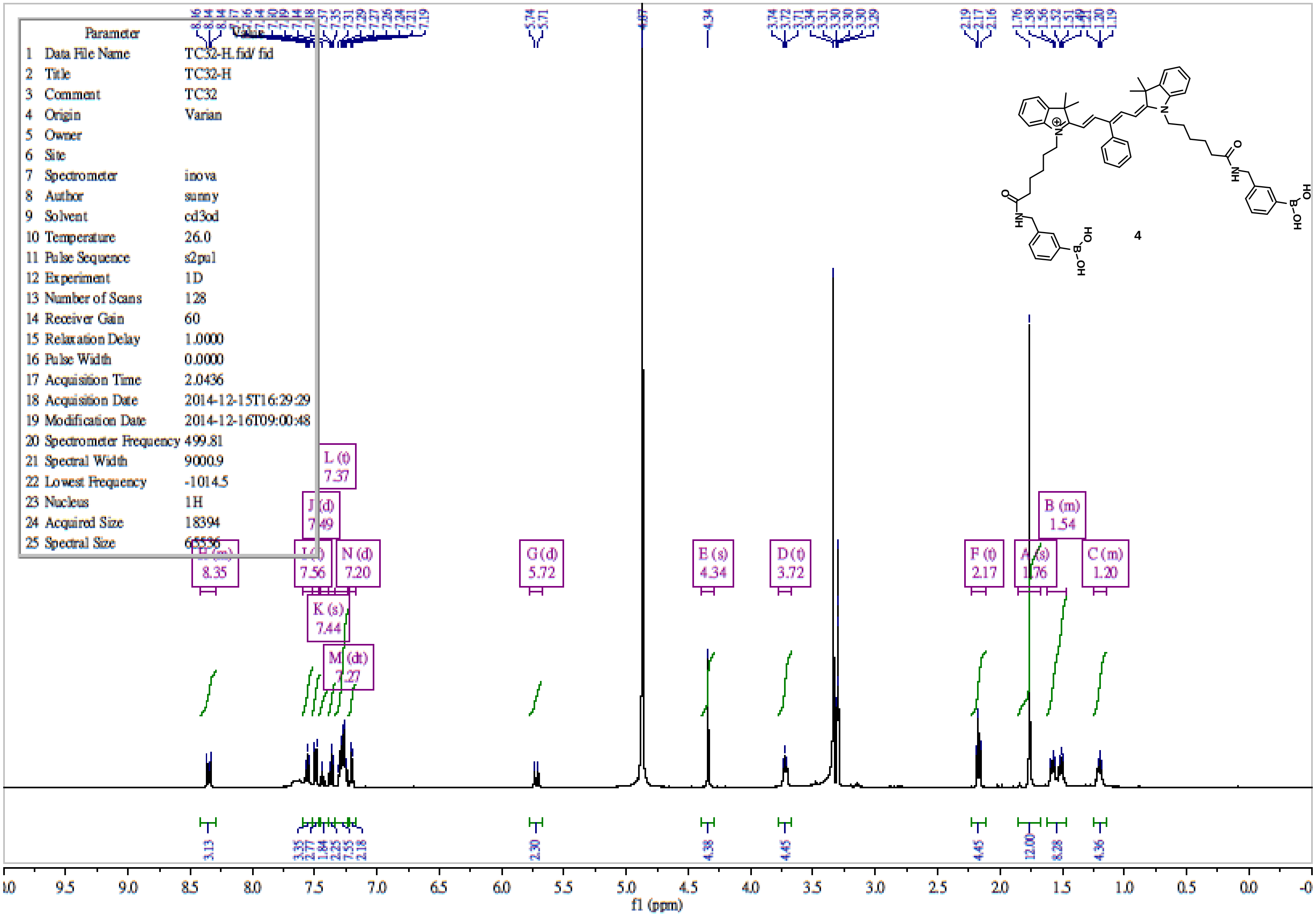
Parameter Value
1 Data File Name TC32-H.fid/ fid
2 Title TC32-H
3 Comment TC32
4 Origin Varian
5 Owner
6 Site
7 Spectrometer inova
8 Author sunny
9 Solvent cd3od
10 Temperature 26.0
11 Pulse Sequence s2pul
12 Experiment 1D
13 Number of Scans 128
14 Receiver Gain 60
15 Relaxation Delay 1.0000
16 Pulse Width 0.0000
17 Acquisition Time 2.0436
18 Acquisition Date 2014-12-15T16:29:29
19 Modification Date 2014-12-16T09:00:48
20 Spectrometer Frequency 499.81
21 Spectral Width 9000.9
22 Lowest Frequency -1014.5
23 Nucleus 1H
24 Acquired Size 18394
25 Spectral Size 65536
4
L (t) 7.37
J (d) 7.49
N (d) 7.20
K (s) 7.44
M (dt) 7.27
G (d) 5.72
E (s) 4.34
D (t) 3.72
F (t) 2.17
B (m) 1.54
A (s) 1.76
C (m) 1.20
f1 (ppm)

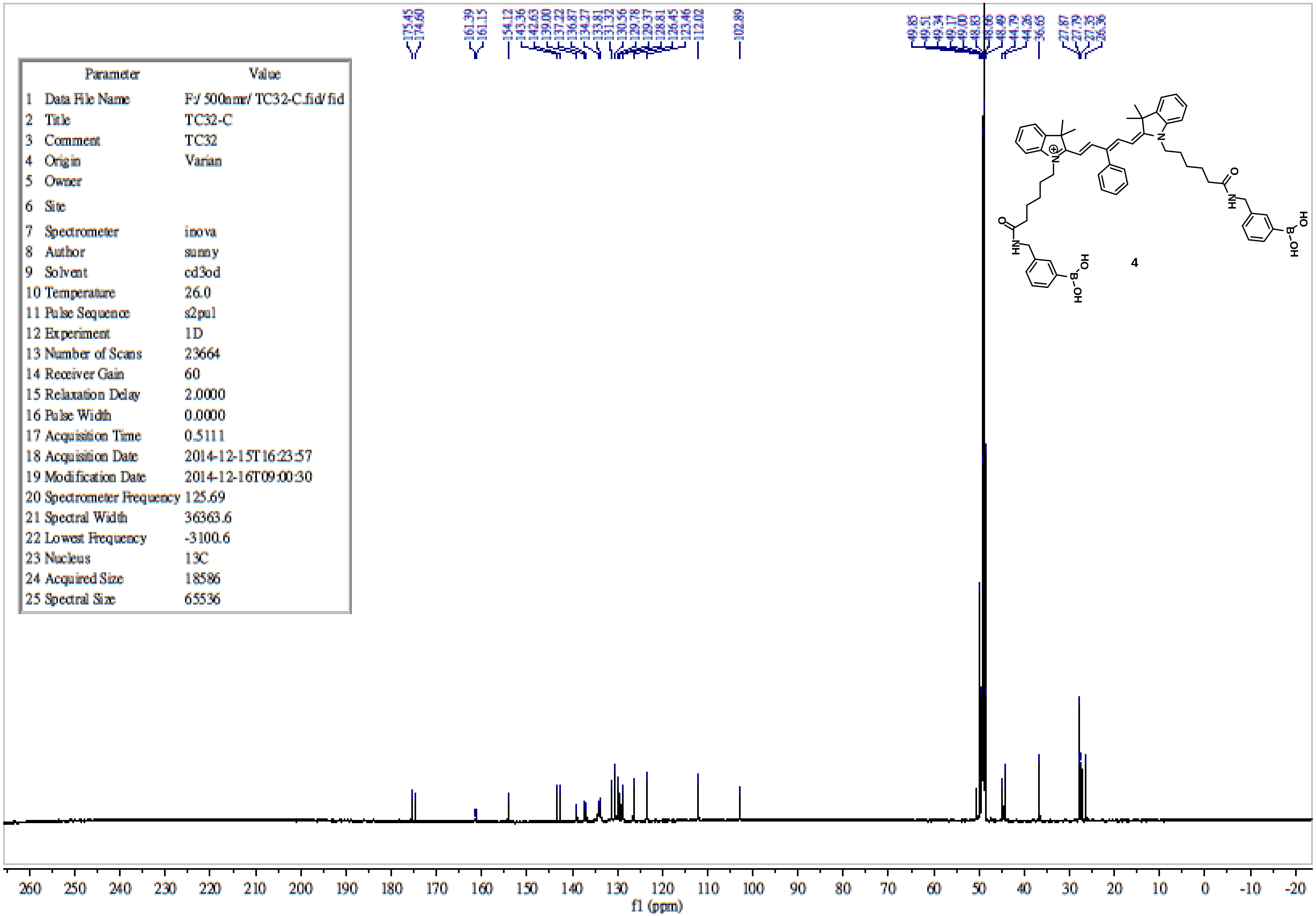

	Parameter	Value
1	Data File Name	F:/ 500nmr/ TC32-C.fid/ fid
2	Title	TC32-C
3	Comment	TC32
4	Origin	Varian
5	Owner	
6	Site	
7	Spectrometer	inova
8	Author	sunny
9	Solvent	cd3od
10	Temperature	26.0
11	Pulse Sequence	s2pul
12	Experiment	1D
13	Number of Scans	23664
14	Receiver Gain	60
15	Relaxation Delay	2.0000
16	Pulse Width	0.0000
17	Acquisition Time	0.5111
18	Acquisition Date	2014-12-15T16:23:57
19	Modification Date	2014-12-16T09:00:30
20	Spectrometer Frequency	125.69
21	Spectral Width	36363.6
22	Lowest Frequency	-3100.6
23	Nucleus	13C
24	Acquired Size	18586
25	Spectral Size	65536

Parameter		Value
1	Title	PI65
2	Comment	PI65
3	Origin	UXNMR, Bruker Analytische Messtechnik GmbH
4	Owner	root
5	Site	
6	Spectrometer	spect
7	Author	
8	Solvent	MeOD
9	Temperature	300.0
10	Pulse Sequence	zg30
11	Experiment	1D
12	Number of Scans	9
13	Receiver Gain	512
14	Relaxation Delay	2.0000
15	Pulse Width	10.0000
16	Acquisition Time	2.5560
17	Acquisition Date	2013-08-08T15:27:00
18	Modification Date	2013-08-08T15:27:56
19	Spectrometer Frequency	400.15
20	Spectral Width	6410.3
21	Lowest Frequency	-414.9
22	Nucleus	1H
23	Acquired Size	16384
24	Spectral Size	32768

^{1}H NMR (400 MHz, MeOD) δ 8.64 (d, J = 9.8 Hz, 2H), 7.47 (t, J = 7.9 Hz, 4H), 7.36 (d, J = 7.8 Hz, 4H), 7.29 (t, J = 7.3 Hz, 2H), 6.23 (t, J = 11.6 Hz, 1H).

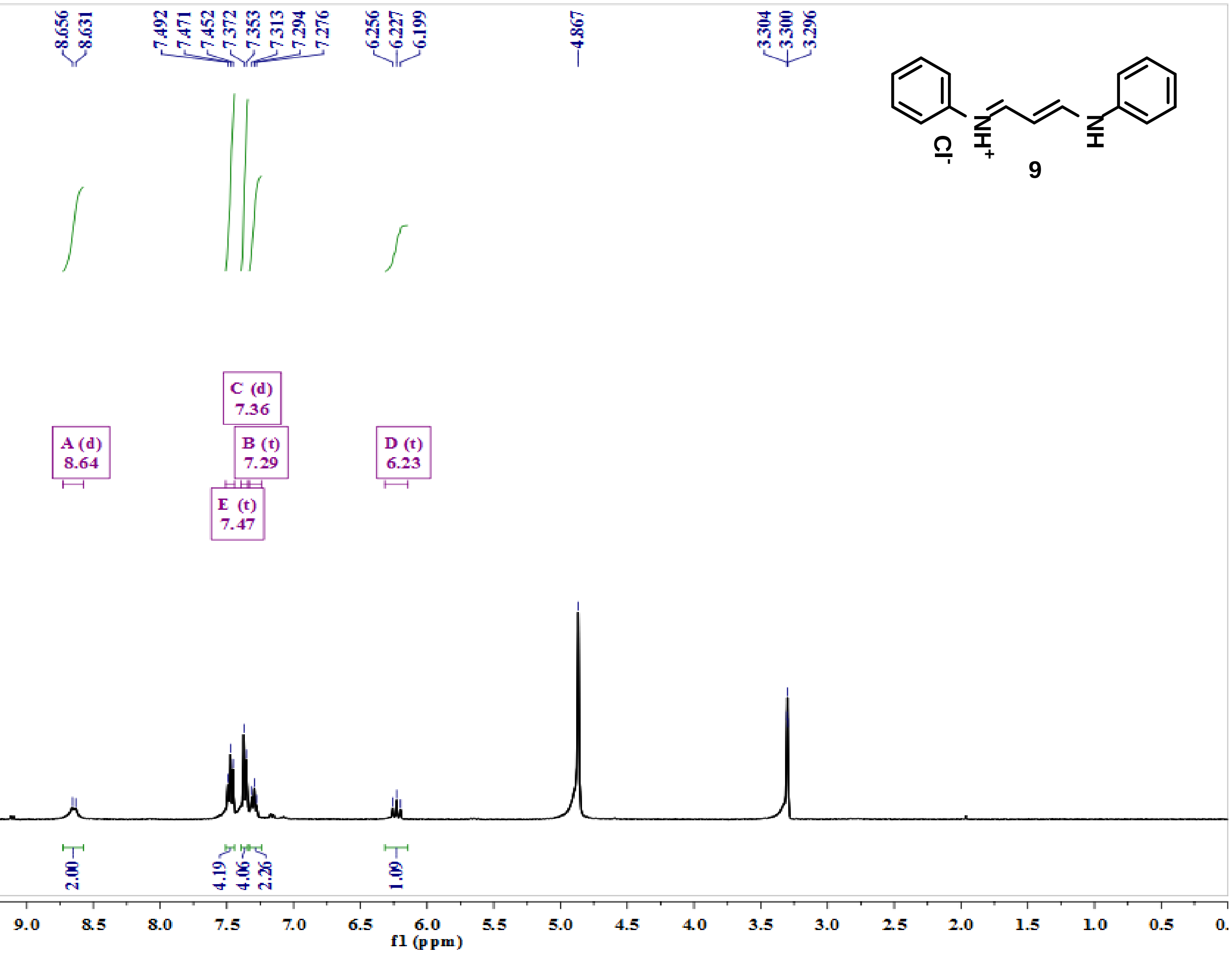

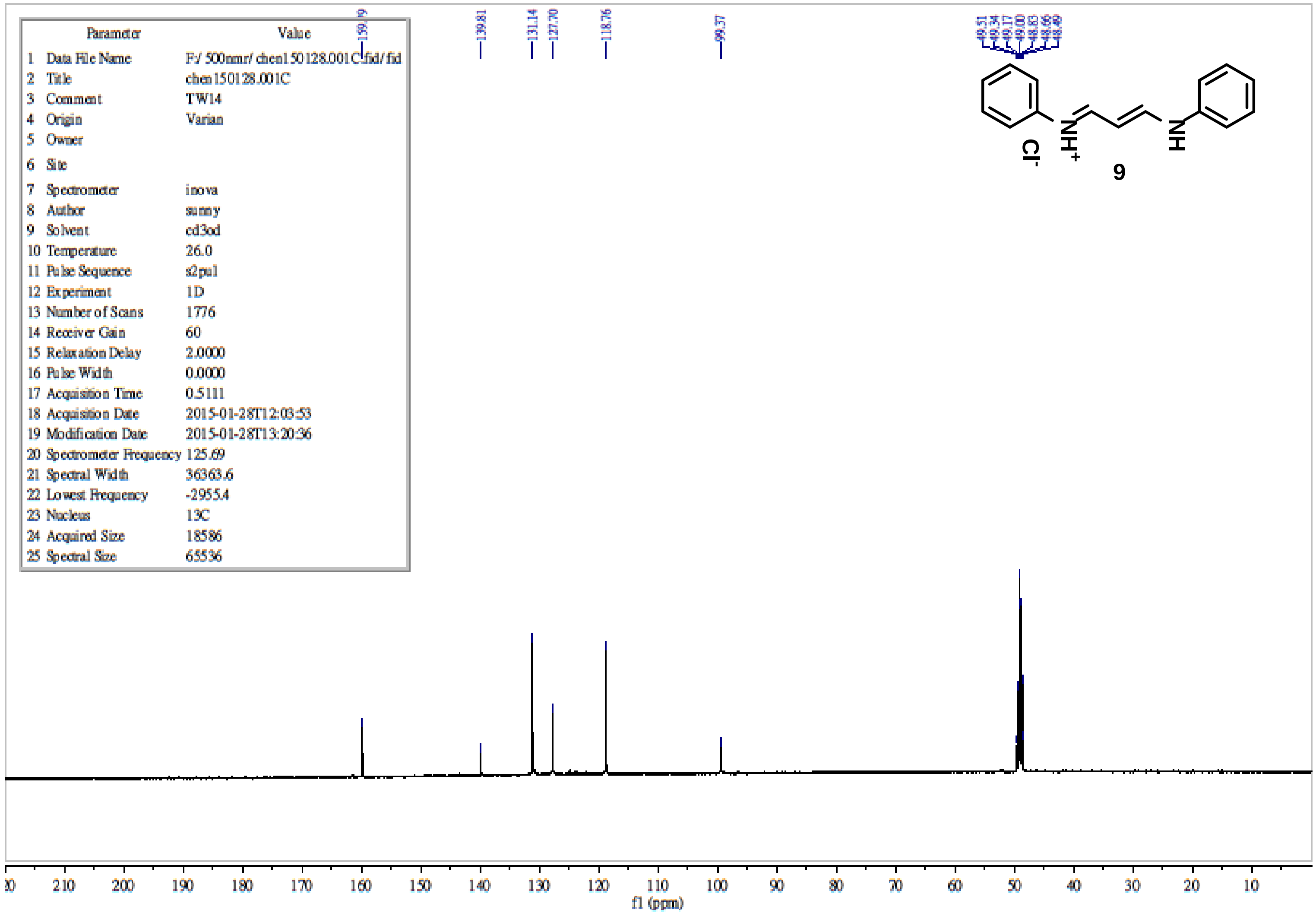

	Parameter	Value
1	Data File Name	F:/ 500nmr/ chen150128.001C.fid/fid
2	Title	chen150128.001C
3	Comment	TW14
4	Origin	Varian
5	Owner	
6	Site	
7	Spectrometer	inova
8	Author	sunny
9	Solvent	cd3od
10	Temperature	26.0
11	Pulse Sequence	s2pul
12	Experiment	1D
13	Number of Scans	1776
14	Receiver Gain	60
15	Relaxation Delay	2.0000
16	Pulse Width	0.0000
17	Acquisition Time	0.5111
18	Acquisition Date	2015-01-28T12:03:53
19	Modification Date	2015-01-28T13:20:36
20	Spectrometer Frequency	125.69
21	Spectral Width	36363.6
22	Lowest Frequency	-2955.4
23	Nucleus	13C
24	Acquired Size	18586
25	Spectral Size	65536

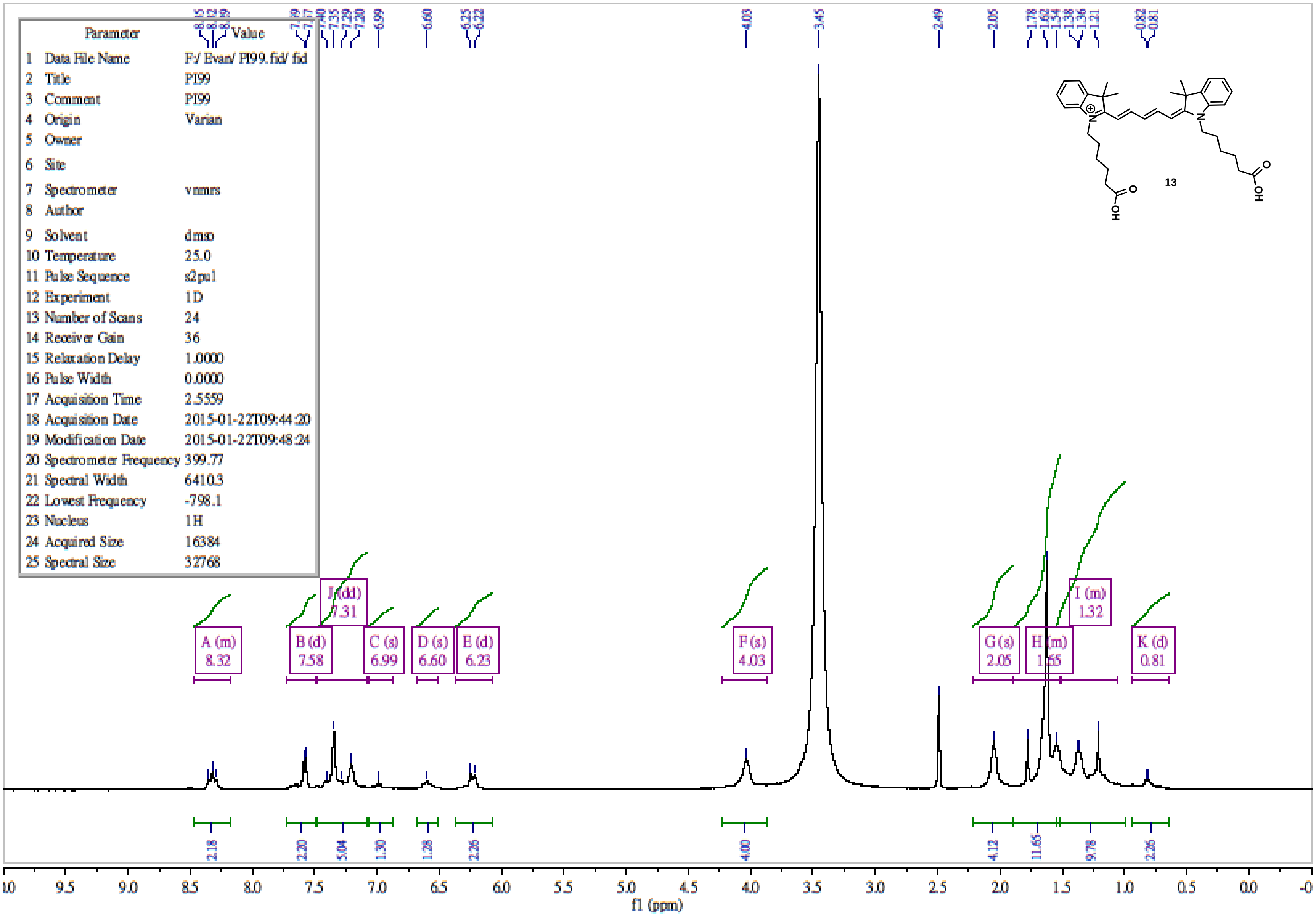

	Parameter	Value
1	Data File Name	F:/ Evan/ PI99.fid/ fid
2	Title	PI99
3	Comment	PI99
4	Origin	Varian
5	Owner	
6	Site	
7	Spectrometer	vnmrs
8	Author	
9	Solvent	dmso
10	Temperature	25.0
11	Pulse Sequence	s2pul
12	Experiment	1D
13	Number of Scans	24
14	Receiver Gain	36
15	Relaxation Delay	1.0000
16	Pulse Width	0.0000
17	Acquisition Time	2.5559
18	Acquisition Date	2015-01-22T09:44:20
19	Modification Date	2015-01-22T09:48:24
20	Spectrometer Frequency	399.77
21	Spectral Width	6410.3
22	Lowest Frequency	-798.1
23	Nucleus	1H
24	Acquired Size	16384
25	Spectral Size	32768

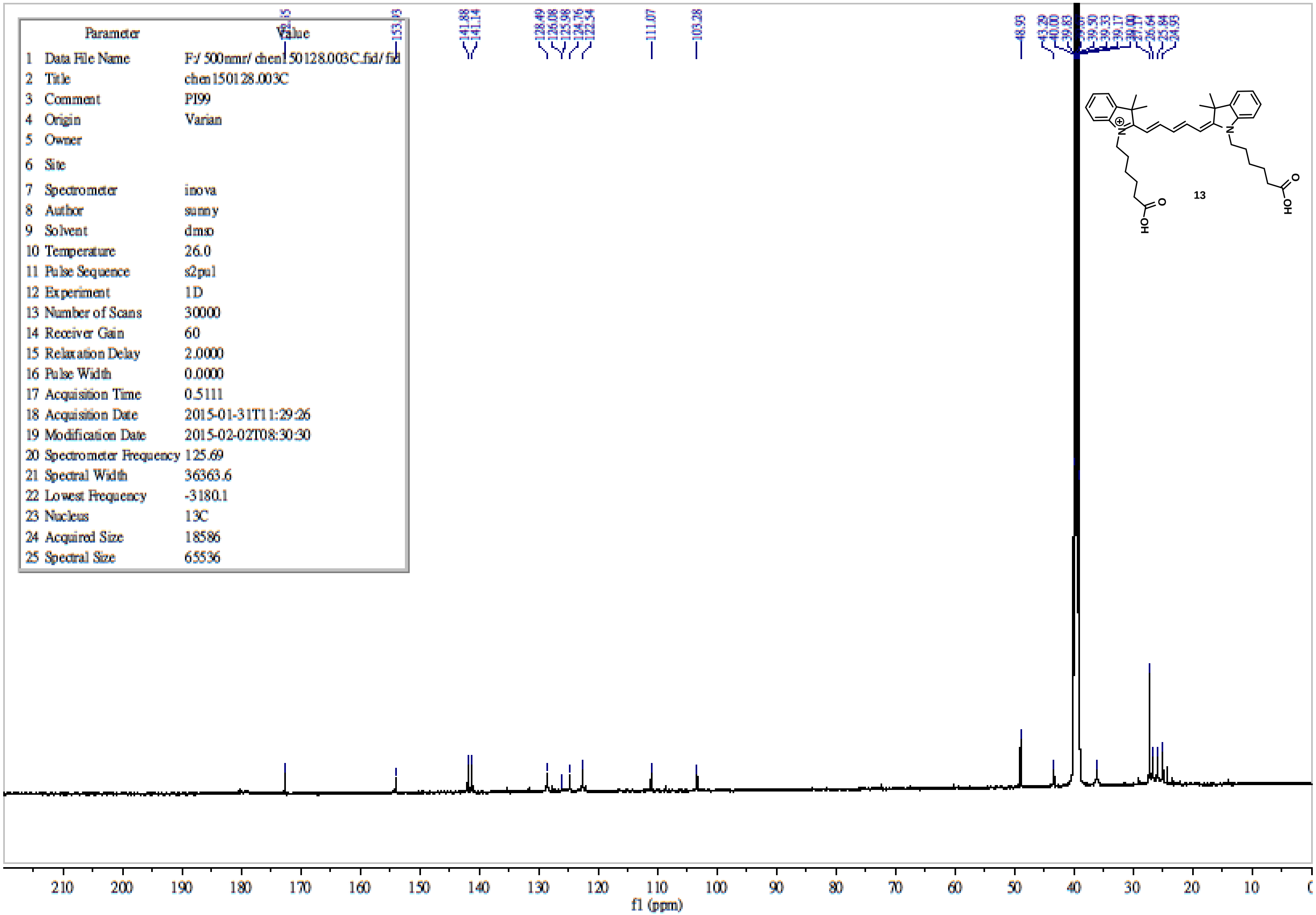

	Parameter	Value
1	Data File Name	F:/ 500nmr/ chen150128.003C.fid/ fid
2	Title	chen150128.003C
3	Comment	PI99
4	Origin	Varian
5	Owner	
6	Site	
7	Spectrometer	inova
8	Author	sunny
9	Solvent	dmso
10	Temperature	26.0
11	Pulse Sequence	s2pul
12	Experiment	1D
13	Number of Scans	30000
14	Receiver Gain	60
15	Relaxation Delay	2.0000
16	Pulse Width	0.0000
17	Acquisition Time	0.5111
18	Acquisition Date	2015-01-31T11:29:26
19	Modification Date	2015-02-02T08:30:30
20	Spectrometer Frequency	125.69
21	Spectral Width	36363.6
22	Lowest Frequency	-3180.1
23	Nucleus	13C
24	Acquired Size	18586
25	Spectral Size	65536

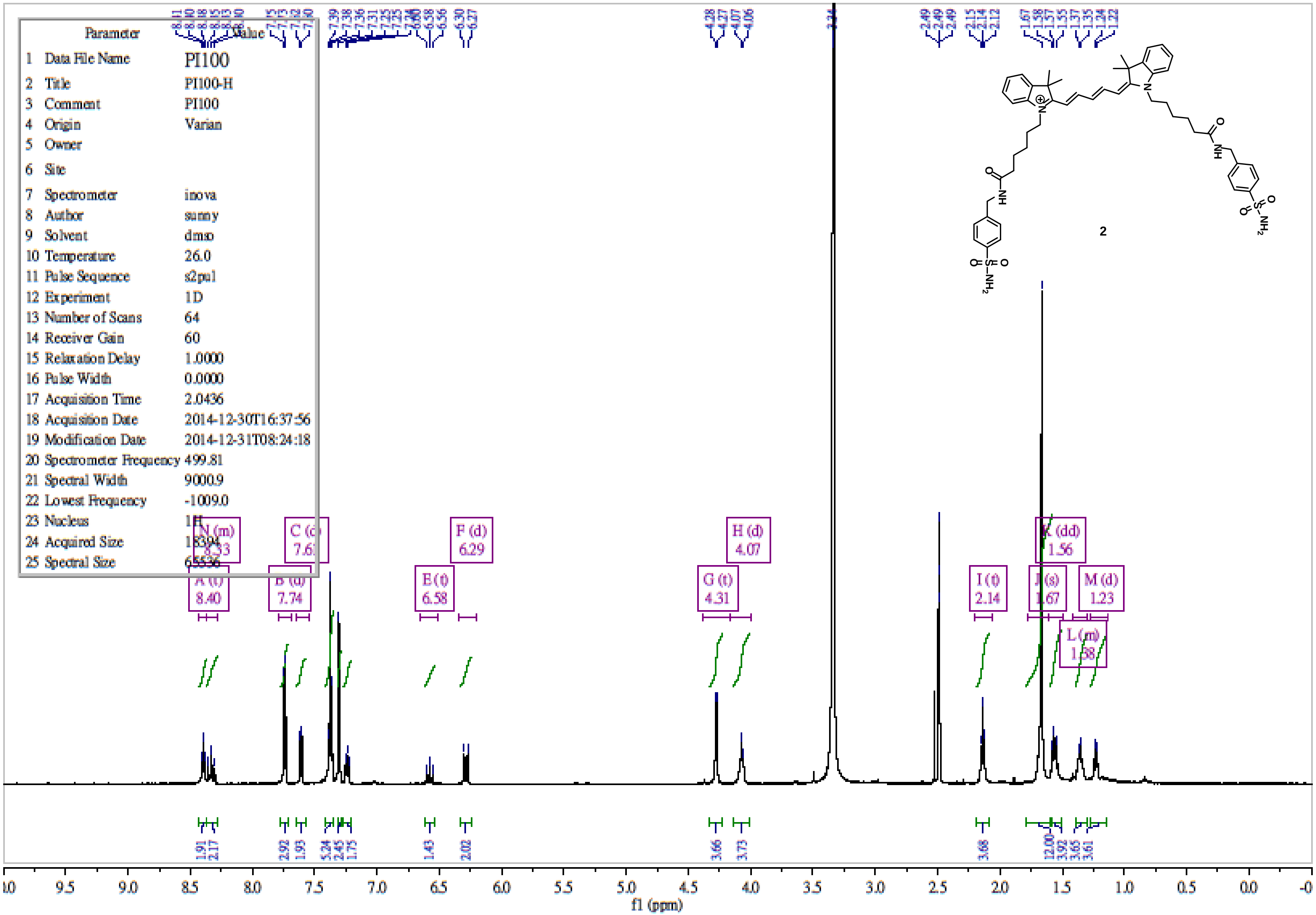

2
Parameter
Value
1 Data File Name PI100
2 Title PI100-H
3 Comment PI100
4 Origin Varian
5 Owner
6 Site
7 Spectrometer inova
8 Author sunny
9 Solvent dmso
10 Temperature 26.0
11 Pulse Sequence s2pul
12 Experiment 1D
13 Number of Scans 64
14 Receiver Gain 60
15 Relaxation Delay 1.0000
16 Pulse Width 0.0000
17 Acquisition Time 2.0436
18 Acquisition Date 2014-12-30T16:37:56
19 Modification Date 2014-12-31T08:24:18
20 Spectrometer Frequency 499.81
21 Spectral Width 9000.9
22 Lowest Frequency -1009.0
23 Nucleus 1H
24 Acquired Size 18394
25 Spectral Size 65536
A (t) 8.40
B (d) 7.74
E (t) 6.58
F (d) 6.29
G (t) 4.31
H (d) 4.07
I (t) 2.14
J (s) 1.67
K (dd) 1.56
L (m) 1.38
M (d) 1.23
f1 (ppm)

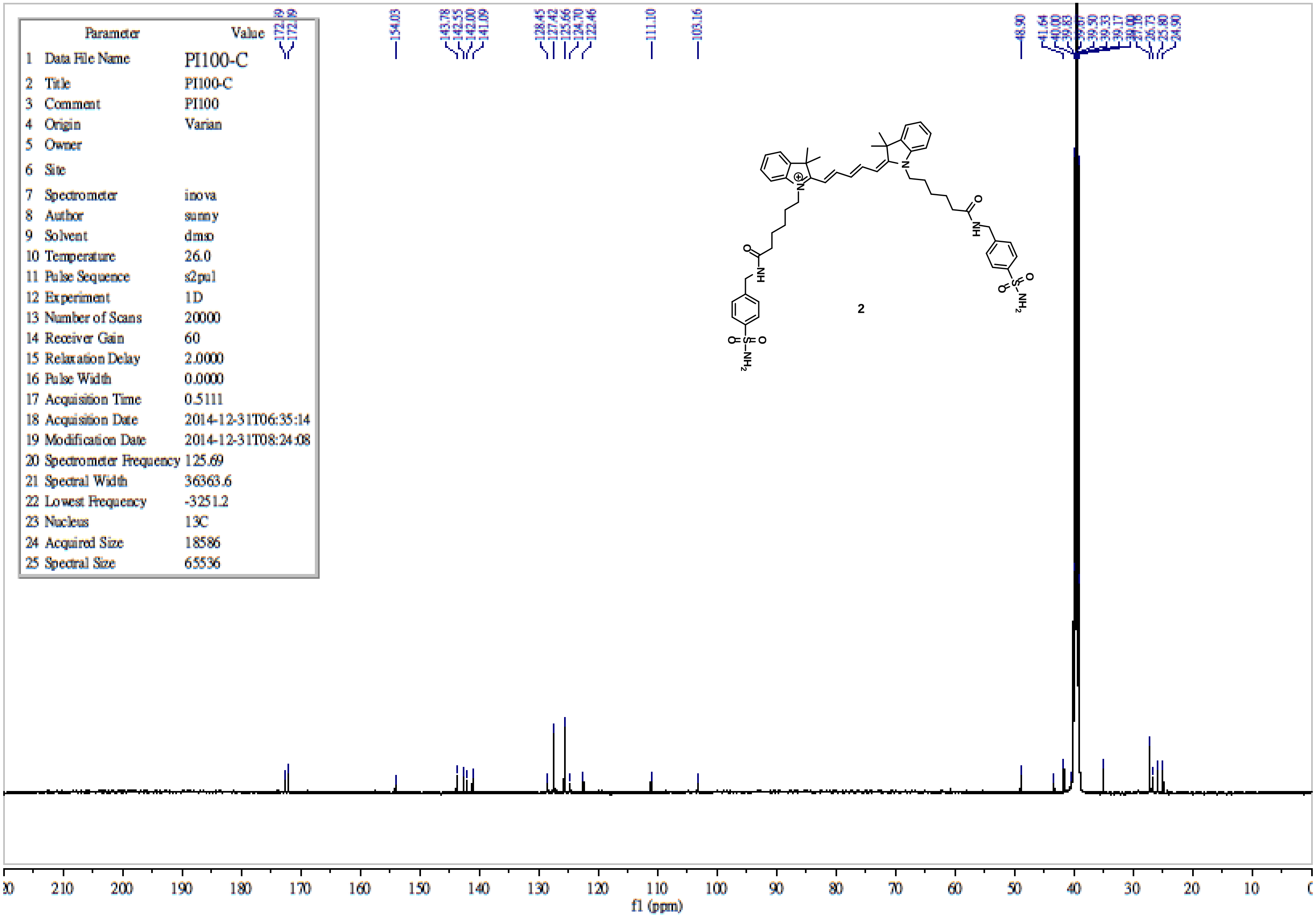

Parameter	Value
1 Data File Name	PI100-C
2 Title	PI100-C
3 Comment	PI100
4 Origin	Varian
5 Owner	
6 Site	
7 Spectrometer	inova
8 Author	sunny
9 Solvent	dmso
10 Temperature	26.0
11 Pulse Sequence	s2pul
12 Experiment	1D
13 Number of Scans	20000
14 Receiver Gain	60
15 Relaxation Delay	2.0000
16 Pulse Width	0.0000
17 Acquisition Time	0.5111
18 Acquisition Date	2014-12-31T06:35:14
19 Modification Date	2014-12-31T08:24:08
20 Spectrometer Frequency	125.69
21 Spectral Width	36363.6
22 Lowest Frequency	-3251.2
23 Nucleus	13C
24 Acquired Size	18586
25 Spectral Size	65536
